# Corticosteroids and Regional Variations in Thickness of the Human Cerebral Cortex across the Lifespan

**DOI:** 10.1093/cercor/bhz108

**Published:** 2019-06-26

**Authors:** Nadine Parker, Didac Vidal-Pineiro, Leon French, Jean Shin, Hieab H H Adams, Henry Brodaty, Simon R Cox, Ian J Deary, Anders M Fjell, Stefan Frenzel, Hans Grabe, Norbert Hosten, Mohammad Arfan Ikram, Jiyang Jiang, Maria J Knol, Bernard Mazoyer, Aniket Mishra, Perminder S Sachdev, Giovanni Salum, Claudia L Satizabal, Helena Schmidt, Reinhold Schmidt, Sudha Seshadri, Gunter Schumann, Henry Völzke, Kristine B Walhovd, Wei Wen, Katharina Wittfeld, Qiong Yang, Stephanie Debette, Zdenka Pausova, Tomáš Paus

**Affiliations:** 1 Institute of Medical Science, University of Toronto, Toronto M5S 1A8, Canada; 2 Bloorview Research Institute, Holland Bloorview Kids Rehabilitation Hospital, Toronto M4G 1R8, Canada; 3 Centre for Lifespan Changes in Brain and Cognition, Department of Psychology, University of Oslo, Oslo 0373, Norway; 4 Centre for Addiction and Mental Health, University of Toronto, Toronto M5T 1L8, Canada; 5 The Hospital for Sick Children, University of Toronto, Toronto M5G 0A4, Canada; 6 Department of Epidemiology, Erasmus MC University Medical Center Rotterdam, Rotterdam 3015, the Netherlands; 7 Department of Radiology and Nuclear Medicine, Erasmus MC University Medical Center Rotterdam, Rotterdam 3015, the Netherlands; 8 Centre for Healthy Brain Ageing (CHeBA), School of Psychiatry, University of New South Wales, Sydney, NSW 2052, Australia; 9 Centre for Cognitive Ageing and Cognitive Epidemiology, University of Edinburgh, Edinburgh EH8 9JZ, UK; 10 Department of Psychology, University of Edinburgh, Edinburg EH8 9JZ, UK; 11 Department of Radiology and Nuclear Medicine, Oslo University Hospital, Oslo 0318, Norway; 12 Department of Psychiatry and Psychotherapy, University Medicine Greifswald, Greifswald 17489, Germany; 13 German Center for Neurodegenerative Diseases (DZNE), Site Rostock/ Greifswald 18147, Germany; 14 Institute for Diagnostic Radiology and Neuroradiology, University Medicine Greifswald, Greifswald 17489, Germany; 15 Groupe d’Imagerie Neurofonctionnelle, Institut des Maladies Neurodégénératives, Centre National de la Recherche Scientifique, Commissariat à l’Energie Atomique, et Université de Bordeaux, Bordeaux 5293, France; 16 Bordeaux Population Health Research Center, INSERM UMR, University of Bordeaux, Bordeaux 33076, France; 17 Neuropsychiatric Institute, Prince of Wales Hospital, Sydney, NSW 2031, Australia; 18 Department of Psychiatry, Federal University of Rio Grande do Sul, Porto Alegre 90040-060, Brazil; 19 National Institute of Developmental Psychiatry for Children and Adolescents (INCT-CNPq), São Paulo, Brazil; 20 Glenn Biggs Institute for Alzheimer's & Neurodegenerative Diseases, UT Health San Antonio, TX 78229, USA; 21 Department of Neurology, Boston University School of Medicine, MA 02118, USA; 22 Gottfried Schatz Research Center for Cell Signaling, Metabolism and Aging, Medical University of Graz 8036, Austria; 23 Clinical Division of Neurogeriatrics, Department of Neurology, Medical University of Graz 8036, Austria; 24 MRC-Social Genetic and Developmental Psychiatry Centre, Institute of Psychiatry, King’s College London, London SE5 8AF, UK; 25 Department of SHIP/Clinical-Epidemiological Research, Institute for Community Medicine, University Medicine Greifswald, Greifswald 17489, Germany; 26 DZHK (German Centre for Cardiovascular Research), Partner Site Greifswald 13316, Germany; 27 DZD (German Centre for Diabetes Research), Site Greifswald 85764, Germany; 28 Department of Biostatistics, Boston University School of Public Health, MA 02118, USA; 29 Department of Neurology, CHU de Bordeaux, Bordeaux 33000, France; 30 Departments of Psychology and Psychiatry, University of Toronto M5T 1R8, Canada; 31 Centre for Healthy Brain Ageing and Dementia Centre for Research Collaboration, University of New South Wales, Sydney, NSW 2025, Australia

## Abstract

Exposures to life stressors accumulate across the lifespan, with possible impact on brain health. Little is known, however, about the mechanisms mediating age-related changes in brain structure. We use a lifespan sample of participants (*n* = 21 251; 4–97 years) to investigate the relationship between the thickness of cerebral cortex and the expression of the glucocorticoid- and the mineralocorticoid-receptor genes (*NR3C1* and *NR3C2*, respectively), obtained from the Allen Human Brain Atlas. In all participants, cortical thickness correlated negatively with the expression of both *NR3C1* and *NR3C2* across 34 cortical regions. The magnitude of this correlation varied across the lifespan. From childhood through early adulthood, the profile similarity (between *NR3C1*/*NR3C2* expression and thickness) increased with age. Conversely, both profile similarities decreased with age in late life. These variations do not reflect age-related changes in *NR3C1* and *NR3C2* expression, as observed in 5 databases of gene expression in the human cerebral cortex (502 donors). Based on the co-expression of *NR3C1* (and *NR3C2*) with genes specific to neural cell types, we determine the potential involvement of microglia, astrocytes, and CA1 pyramidal cells in mediating the relationship between corticosteroid exposure and cortical thickness. Therefore, corticosteroids may influence brain structure to a variable degree throughout life.

## Introduction

Throughout the human lifespan, exposures to life stressors accumulate with possible consequences for brain structure and function, including mental health. Cumulative exposure to stress and passing of time, namely aging, are intimately linked. Stress has been associated with accelerated aging particularly in the form of telomere shortening and age-related epigenetic modifications (Epel et al. 2004; Shalev et al. 2013; Haussmann and Heidinger 2015; Gassen et al. 2017). Additionally, aging may result in a dysregulation of the hypothalamic-adrenal pituitary (HPA) axis and reduced ability to adapt to stressors; this phenomenon is commonly marked by a prolonged recovery time in older adults (Sapolsky et al. 1986; Ferrari et al. 2001; Seeman et al. 2001).

Glucocorticoids, the main mediators of the stress response, are a class of corticosteroids with intracellular receptors capable of both genomic (i.e., transcriptional) and non-genomic (e.g., membrane associated) activity (Prager and Johnson 2009; Menke et al. 2012; Liston et al. 2013; Niwa et al. 2013; Arloth et al. 2015; Gassen et al. 2017; Zannas and Chrousos 2017). Within the brain, there are 2 highly homologous corticosteroid receptors through which glucocorticoids act: the abundant glucocorticoid receptor (GR), encoded by the *NR3C1* gene, and the less abundant mineralocorticoid receptor (MR), encoded by the *NR3C2* gene (Arriza et al. 1988). It is known that MR has a 10-times greater affinity for glucocorticoids than GR (Reul and De Kloet 1985; Arriza et al. 1988). This difference in receptor affinity results in a dose effect whereby MR and GR are occupied, respectively, at low and high glucocorticoid levels. Although highly lipophilic, the passage of corticosteroids across the blood brain barrier is partially inhibited by the efflux transporter, P-glycoprotein (Geerling and Loewy 2009). The crossing of some corticosteroids (e.g., cortisol and aldosterone) is more hindered than others (e.g., corticosterone; Geerling and Loewy 2009). Due to higher concentrations, glucocorticoids outcompete mineralocorticoids for MR binding in brain tissue (Yongue and Roy 1987; Arriza et al. 1988; Geerling and Loewy 2009). Moreover, MR is thought to be constitutively occupied with high nuclear concentration even at basal circulating levels of corticosteroids (Reul and De Kloet 1985; Gesing et al. 2001). Activation of MR increases neuronal excitability and elicits anti-apoptotic/neuroprotective cellular effects. Conversely, GR activation can decrease neuronal excitability and cause pro-apoptotic/deleterious cellular effects (Almeida et al. 2000; Crochemore et al. 2005; Prager and Johnson 2009; Sapolsky 2015). Thus, acting via ligand-bound corticosteroid receptors, glucocorticoid exposure may induce changes in brain structure detectable with magnetic resonance imaging (MRI).

**Table 1:** Cohort and gene expression source characteristics

**Cohort Participant Demographics**			
**Cohort**	**Participants (*n*)**	**Median age (range)**	**Sex (%Male)**
HRC^a,c^	1107 (454)	12.0 (6–18)	60.8
SYS (Adolescents)^d^	987	14.8 (12–19)	48.5
IMAGEN^a,d^	1823 (1203)	14.9 (12–22)	47.7
BIL&GIN^d^	453	24.0 (18–56)	48.8
HCP^e^	1200	29 (22–37)	45.6
LCBC^a,c^	1755 (986)	36.6 (4–93)	40.5
SYS (Parents)^d^	541	49.7 (36–65)	44.9
SHIP&TREND^d^	3021	52.9 (21–90)	47.3
DLBS^e^	315	54.3 (20–89)	37.1
RS^b,d^	5720 (11 600 sim.)	63.0 (45–100) [64.1 (46–94) sim]	44.6
SALD^e^	494	64.0 (19–80)	37.9
MAS^d^	358	64.5 (25–97)	28.9
FHS^b,d^	999 (1000 sim.)	64.0 (41–93) [64.1 (41–92) sim]	41.8
ASPS_Fam^d^	332	68.1 (38–84)	39.2
OATS^d^	403	69.2 (65–97)	34.5
OASIS^a,e^	671 (389)	69.5 (42–95)	39.5
3C_Dijon^d^	436	72.2 (65–83)	42.0
LBC1936^d^	636	72.7 (71–74)	52.8
**Gene Expression Databases**			
**Source**	**Donors (*n*)**	**Median Age (range)**	**Sex (%Male)**
Allen Human Brain Atlas	6	42.5 (24–57)	83.3
BrainCloud	206	36.3 (4–78)	68.5
BrainEAC	134	57.9 (16–102)	73.9
BrainSpan	14	20.2 (4–40)	57.1
GTEx	142	57.9 (25–80)	69.7

HRC, Brazilian High Risk Cohort for Psychiatric Disorders; SYS, Saguenay Youth Study; BIL&GIN, Brain Imaging of Lateralization by the Groupe d’Imagerie Fonctionnelle; LCBC, Lifespan Changes in Brain and Cognition; HCP, Human Connectome Project; SHIP&TREND, Study of Health in Pomerania and SHIP-TREND; DLBS, Dallas Lifespan Brain Study; RS, Rotterdam Study; SALD, Southwest University Adult Lifespan Dataset; MAS, Memory and Aging Study; FHS, Framingham Heart Study; ASPS_Fam, Austrian Stroke Prevention Study Family Cohort; OATS, Older Australian Twin Study; OASIS, Open Access Series of Imaging Studies; 3C_Dijon, The 3 City Study—Dijon location; LBC1936, Lothian Birth Cohort.

a ^a^Longitudinal studies where participants *n* in brackets (column 2) represent the subsample of participants with more than 1 scan.

b ^b^Simulated data were used for both the Rotterdam and Framingham study. Therefore, number of participants and median age (and ranges) will have both an observed sample value and a simulated value. Sim, based on simulated data.

c ^c^Collaborating cohort outside of the BRIDGET Consortium.

d ^d^Cohorts included in the BRIDGET Consortium.

e ^e^Cohorts with open access data.

The variation in corticosteroid receptor gene expression may result in differing susceptibility to glucocorticoid exposure. In a previous study of the adolescent brain, we showed that age-related decreases in cortical thickness were negatively correlated with the expression of *NR3C1* across 34 regions of the cerebral cortex (Wong et al. 2017). We have also reported that this relationship appears stronger in female adolescents living in a potentially stressful social environment (Parker et al. 2017). It is well established that cortical thinning occurs during adolescence and continues into adulthood (Sowell et al. 2007; Tamnes et al. 2010). This cortical thinning may, in part, be due to age-related reductions in dendritic and synaptic complexity (Huttenlocher 1979; Nakamura et al. 1985; Kolb et al. 2003; Selemon 2013; Koss et al. 2014), a structural modification also linked to glucocorticoid exposure (Vyas et al. 2002; Cook and Wellman 2004; Liston and Gan 2011; Liston et al. 2013). Therefore, typically developing adolescents may exhibit glucocorticoid-associated changes in cortical thickness. It is still unknown if this association exists outside of the adolescent period.

The present investigation aims to examine the lifespan association between corticosteroids and brain structure, namely cortical thickness, using inter-regional variations in cortical expression of *NR3C1* and *NR3C2* as a proxy of inter-regional variations in response to corticosteroids. Here we use a large, multi-cohort, lifespan sample (*n* = 21 251; age 4–97 years) of individuals to investigate the relationship between the inter-regional profile of cortical thickness, estimated from MRI, and the inter-regional profile of *NR3C1* and *NR3C2* expression, obtained from the Allen Human Brain Atlas. With cumulative exposure to glucocorticoids with aging, we hypothesize that cortical thickness may become more strongly associated with the distribution of *NR3C1* and *NR3C2* cortical expression across the lifespan. Furthermore, we investigate neural cell types that may be involved in mediating the relationship between glucocorticoid exposure and cortical thickness. Finally, we examine whether expression of *NR3C1* and *NR3C2* in the human cerebral cortex varies across the lifespan, using data from 502 post-mortem donor brains (4–102 years of age).

## Materials and Methods

### Participants

A group of 18 international cohorts (14 cross-sectional), with a combined total of 21 251 participants (54.9% female), contributed data for this study. All cohorts contributed demographic (age and sex) and neuroimaging data from cognitively healthy participants across the lifespan (age range, 4–97 years). All included participants had at least 1 T1-weighted brain scan. Basic demographic and cohort details can be found in Table 1.

### Neuroimaging

For each participant, cortical thickness was estimated for 34 cortical regions (left hemisphere) using the FreeSurfer pipeline with the Desikan–Killiany Atlas (Desikan et al. 2006). We used left hemisphere values of cortical thickness to ensure compatibility with gene expression data available in the Allen Human Brain Atlas (left hemisphere only, see below). Note that inter-regional profiles of cortical thickness in the right hemisphere are virtually identical to those in the left hemisphere; in a sample of 2091 participants (3514 scans), we observed a strong correlation between the left and right inter-regional profiles (*r* = 0.99). Specifics on MRI protocol and FreeSurfer version can be found in Supplementary Table S1.

### Gene Expression

The Allen Human Brain Atlas provides mRNA expression from multiple regions of the left hemisphere of 6 donor brains (age 24–57 years, 1 female); mRNA was quantified using Agilent micro-arrays, and each brain sample was mapped to MNI coordinates (Supplementary, Hawrylycz et al. 2012; Mazziotta et al. 2001). Using these data, French and Paus (2015) mapped gene expression to the 34 cortical regions parcellated using the Desikan–Killiany Atlas. Briefly, 1) probe expression values (*n* = 58 692) were averaged at the gene level (*n* = 20 737), 2) Freesurfer V5.3 was run on the MNI152 template to map sample coordinates to Desikan–Killiany Atlas regions, with anatomical annotations helping to increase the number of correctly mapped samples, 3) each donor had between 6 and 100 samples per region; therefore, gene expression values were averaged at the regional level resulting in a single expression value for each of the cortical region (*n* = 34), and 4) across the 6 donors, median gene expression was calculated for each brain region. The final result is a single gene expression (median) value for each of the 34 regions.

**Figure 1:**
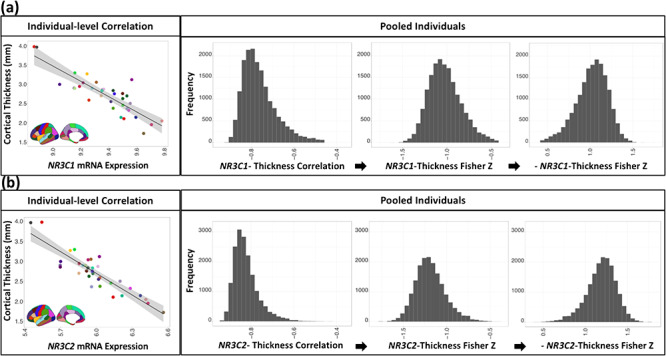
Creation of the *NR3C1*- (*a*) and *NR3C2*- (*b*) Thickness Similarity measure. Each measure starts from an individual-level correlation between regional cortical thickness (in each of the 34 regions) and gene expression. Since gene expression is obtained from the Allen Human Brain Atlas, these values are constant across all individuals. The individual-level correlations are pooled, Fisher-Z transformed, and then multiplied by negative 1 (inverted).

Given the lifespan nature of our study, we examined whether *NR3C1* and *NR3C2* expression varies with age. To do so, we obtained mRNA expression values from the following (additional) sources of gene expression in the human brain: BrainCloud (Supplementary, Colantuoni et al. 2012; Jaffe et al. 2015), Brain eQTL Almanac (BrainEAC; Trabzuni et al. 2011), BrainSpan (Li et al. 2018), and Genotype Tissue Expression (GTEx; Ardlie et al. 2015). Each source acquired tissue samples from human post-mortem donor brains. The extent of tissue sampling ranges from a single cortical region (BrainCloud) to 34 cortical regions (Allen Human Brain Atlas). When applicable, we excluded donors younger than 4 years of age (the youngest age in the in vivo MRI dataset). Across these datasets, the age span was from 4 to 102 years. General demographics of each source can be seen in Table 1. A description of each database and information on data acquisition can be found in Supplementary Table S2.

### *NR3C1*- and *NR3C2*-Cortical Thickness Similarity

We assessed the degree of similarity in inter-regional profiles of gene expression (Allen Human Brain Atlas) and inter-regional profiles of cortical thickness (procedure depicted in Fig. 1). For each participant, the 34 regional cortical thickness values were correlated with *NR3C1* expression (Allen Human Brain Atlas). By design, values of cortical thickness varied across participants while values of gene expression remained constant. Next, a Fisher-Z transformation was applied to all correlation coefficients. For ease of interpretation, the Fisher-Z values were then multiplied by negative 1; a higher value represents a greater similarity between inter-regional profiles of cortical thickness and gene expression. The resulting values were then termed *NR3C1-*Thickness Similarity. The same procedure was followed for *NR3C2* resulting in a distribution of *NR3C2*-Thickness Similarities (Fig. 1*b*). It is important to note that variations in the *NR3C1-* and *NR3C2-*Thickness Similarity are solely due to individual variations in the inter-regional profiles of cortical thickness. The same (reference) values of *NR3C1* and *NR3C2* expression (Table S3) are used in all individual-level correlations between cortical thickness and gene expression across the 34 cortical regions.

For the Framingham Heart Study (FHS) and the Rotterdam Study (RS), gene thickness similarities were simulated from sex-stratified and age-binned individual-level values to comply with local data-sharing regulations. The simulation procedure is outlined in Supplementary Table S4. Similarities in simulated and observed gene-thickness similarities were evaluated using Kolmogorov–Smirnov tests of the distributions as well as by a comparison of age coefficients (procedure outlined in Supplementary Table S4). Age coefficients, from fitting observed and simulated data, were highly comparable (Supplementary Table S5; Supplementary Figs S1 and S2).

### Co-expression of *NR3C1* and *NR3C2* Genes with Cell-Specific Genes

We compared co-expression of *NR3C1* and *NR3C2* with gene panels specific to the following 9 neural cell types, as identified by Zeisel et al. (2015): astrocytes, CA1 pyramidal cells, endothelial cells, ependymal cells, interneurons, microglia, mural cells, oligodendrocytes, and S1 pyramidal cells. Included cell-specific genes had to pass a 2-stage filter for reliability of gene expression profiles across the cerebral cortex, as described by Shin et al. (2017). A resampling approach was used to test for significance in co-expression profiles between *NR3C1* (or *NR3C2*) and the panel of genes specific to a cell type. For each cell-specific panel of genes, a random sample, equal in size to the gene-panel, was selected from all the genes in the Allen Human Brain Atlas that passed the 2-stage filtering (*n* = 2511). Expression of each gene in the random sample was then correlated with expression of the gene of interest (*NR3C1* or *NR3C2*) and the mean correlations coefficient was calculated (test statistic). This random sampling and averaging of correlation coefficients was repeated 10 000 times for each panel of genes. The distribution of the resulting mean values, 10000 per 9 cell-specific gene-panels, comprised an empirical null distribution. The proportion of the null distribution of means that exceed the observed mean correlations of *NR3C1* (or *NR3C2*) with the true cell-specific panel genes were used for a 2-sided *P* value at an FDR corrected alpha = 0.05.

### Gene-Thickness Similarities that Increase with Age in Late Life: Post-hoc Analysis

After observing a decline in both *NR3C1*- and *NR3C2*-Thickness Similarities in late life, we investigated which other genes show an age-related increase in association with cortical thickness during the same time-period. In a subsample of participants greater than 68 years old (start of *NR3C1*-Thickness Similarity decline), the profiles of all 2511 genes that pass the 2-stage consistency filtering were correlated with the profile of cortical thickness. Next, we determined which of the gene-thickness similarities increase with age. This was accomplished by calculating the mean of each gene-thickness similarity and the age-associated slope, derived from linear models adjusting for sex and scanner site. There are 2 cases where the gene-thickness similarity would on average increase with age: 1) if the mean gene-thickness similarity was greater than zero and the slope was positive or 2) if the mean gene-thickness similarity was less than zero and the slope was negative. After identifying those gene-thickness similarities that met either of the 2 criteria, we conducted a gene ontology (GO) enrichment analysis. The 2511 genes with consistent cortical profiles were used as the background gene set, and GO group size was restricted to < 300 genes.

### Statistical Analysis

To determine the effect of age on cortical thickness in each of the 34 regions, we used generalized additive mixed models (GAMMs; *mgcv* package in R Wood 2006) with a cubic spline smoothing terms for age. Sex and scanner site were included as fixed effects. GAMM fitting is well suited for estimating non-linear trajectories—such as thickness estimates across the lifespan—and is robust to changes in modeled parameters such as age range (Storsve et al. 2014; Fjell et al. 2015; Vandekar et al. 2015). To adjust for repeated measures and participant kinship, random effects for participant ID and family ID were included. We then tested the association between age and the *NR3C1-* and *NR3C2-*Thickness Similarity with outliers defined as greater than 3 standard deviations (SDs) from the mean. To fit the lifespan trajectories, we again used a GAMM with sex and scanner site included as fixed effects and participant ID and family ID as random effects. The same procedure was used to test the association between age and *NR3C2*-Thickness Similarity. We compared *NR3C1*-Thickness Similarity with *NR3C2*-Thickness Similarity by 1) overall mean and 2) rate of change with age using first derivatives of the GAMM functions (*gratia* package in R), in early (<28 years; peak in *NR3C2*-Thickness Similarity) and late life (>68 years; initial-decline in *NR3C1*-Thickness Similarity). To determine significance of these comparisons, we used bootstrapped test statistics (10 000 iterations) where a Gene-Thickness Similarity label was randomly assigned to each participant prior to calculating the test statistics. Due to limited overlap in sampled regions, *NR3C1* and *NR3C2* expression from all 5 gene expression databases were scaled and pooled by cerebral lobe (frontal, parietal, temporal, and occipital). Within each of the lobes, we used linear mixed effect models with fixed effects for age and sex as well as random effects for donor ID. All statistical analyses were performed using R v3.5.1 (https://www.r-project.org/), and reported significance values (*P* < 0.05) are FDR corrected.

## Results

A total of 21 251 participants, with 30 143 T1-weighted scans, were included in this study. Age ranged from 4.12 to 97.13 years (mean = 49.56, SD = 23.38), and females comprised 54.86% of the sample (Table 1).

### Group-Level Analysis: Cortical Thickness and *NR3C1* (*NR3C2*) Expression

Only studies that provided true estimates of cortical thickness for each individual were included in this group-level analysis. This sample subset included 13 793 participants (45.0% male) with an age range between 4.12 and 97.13 [mean age = 38.41 (±24.05) years]. All 34 cortical regions exhibited age-related variation in thickness across the lifespan (Supplementary Fig. S3; Supplementary Table S6). Across these cortical regions, we observed a strong negative correlation between *NR3C1* expression and the group mean cortical thickness (*R*^2^ = 0.70, *P* < 0.001) as well as *NR3C2* expression and the group mean cortical thickness (*R*^2^ = 0.83, *P* < 0.001; Supplementary Fig. S3).

### Individual-Level Analysis: Cortical Thickness and *NR3C1* (*NR3C2*) Expression

All participants exhibited a negative correlation between cortical thickness and *NR3C1* (*NR3C2*) expression across the 34 cortical regions (Fig. 1). Thus, consistent with the group-level pattern, cortical regions with high *NR3C1* (*NR3C2*) expression are thinner than regions with low expression.

### Age-Related Changes in the *NR3C1* (*NR3C2)* Thickness Similarity

**Figure 2:**
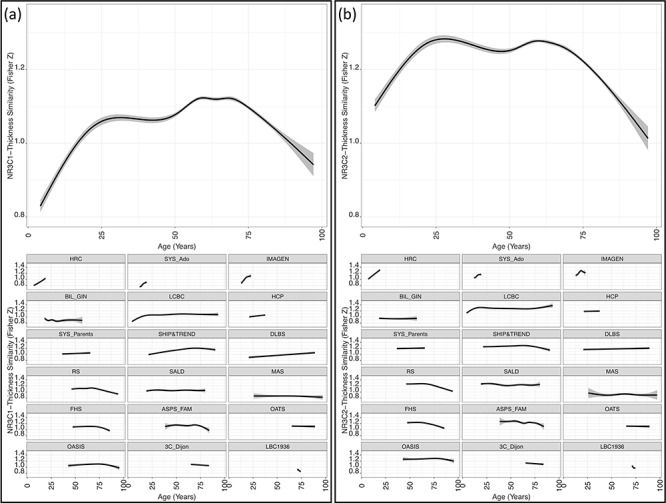
*NR3C1*- and *NR3C2*-Thickness Similarity lifespan trajectories modeled using GAMM analysis with a cubic spline smoother for age. The 95% confidence intervals (CIs) are shown in gray. For visualization with the inclusion of data points see Supplementary Figure S4. Both gene similarities show similar lifespan trajectories. (*a*) Top: The *NR3C1*-Thickness Similarity increases rapidly during the first 2 decades of life before peaking at age 30 years. In contrast, at age 68 years there is a monotonic decline with age. Bottom: *NR3C1*-Thickness Similarity by age modeled in each study separately. Study-specific statistics can be found in Supplementary Table S7. (*b*) The *NR3C2*-Thickness Similarity increases rapidly during the first 2 decades of life before peaking at age 27.9. By age 59.9 years a monotonic decrease in *NR3C2*-Thickness Similarity occurs*.* Bottom: *NR3C2*-Thickness Similarity by age modeled in each study separately. Study-specific statistics can be found in Supplementary Table S7. HRC, Brazilian High Risk Cohort for Psychiatric Disorders; SYS, Saguenay Youth Study; BIL_GIN, Brain Imaging of Lateralization by the Groupe d’Imagerie Fonctionnelle; LCBC, Lifespan Changes in Brain and Cognition; HCP, Human Connectome Project; SHIP&TREND, Study of Health in Pomerania and SHIP-TREND; DLBS, Dallas Lifespan Brain Study; RS, Rotterdam Study; SALD, Southwest University Adult Lifespan Dataset; MAS, Memory and Aging Study; FHS, Framingham Heart Study; ASPS_Fam, Austrian Stroke Prevention Study Family Cohort; OATS, Older Australian Twin Study; OASIS, Open Access Series of Imaging Studies; 3C_Dijon, The 3 City Study - Dijon location; LBC1936, Lothian Birth Cohort 1936.

As explained above, each participant’s correlation coefficient (thickness by gene expression across 34 regions) was Fisher-Z transformed and multiplied by negative 1; a higher value represents a greater similarity between inter-regional profiles of cortical thickness and gene expression. The *NR3C1*-Thickness Similarity varied in strength across the lifespan (*F*_(7,30 143)_ = 243.8, *P* < 0.001; Fig. 2*a*). From childhood through early adulthood, we observe an age-related increase in the *NR3C1*-Thickness Similarity. Therefore, the inter-regional profile of cortical thickness became more similar to the inter-regional profile of *NR3C1* expression. This trend peaked at 30 years after which a relative plateau is observed (Fig. 2*a*). At 68 years, we begin to see a monotonic decrease in *NR3C1*-Thickness Similarity. Thus, in late life, the inter-regional profile in cortical thickness departs from the inter-regional profile of *NR3C1* mRNA expression. Across the lifespan, males had greater *NR3C1*-Thickness Similarity than females (beta = 4.39e-03, *P* = 0.021).

A similar lifespan trajectory was observed for the *NR3C2*-Thickness Similarity (Fig. 2*b*; age, F_(7,30 087)_ = 217.8, *P* < 0.001). Maturation of the cerebral cortex also resembled the distribution of *NR3C2* mRNA expression where *NR3C2*-Thickness Similarity increased in early life (peak at age 27.9 years), plateaued in midlife, and declined after 59.9 years. Females had a greater *NR3C2*-Thickness Similarity than males (beta = 2.01e-02, *P* < 0.001).

### Differences in *NR3C1*- and *NR3C2*-Thickness Similarity

Despite the strong correlation between *NR3C1* and *NR3C2* expression across the 34 cortical regions (*R*^2^ = 0.75, *P* < 0.001), the 2 genes differed in the strength of association with cortical thickness. On average, the *NR3C2*-Thickness Similarity was stronger than the *NR3C1*-Thickness Similarity (mean_difference_ = 0.15, *P* < 0.001) indicating that—across the 34 cortical regions—cortical thickness correlates more strongly with *NR3C2* (than *NR3C1*) expression. The first derivatives of the GAMM lifespan trajectories (Supplementary Fig. S5) were used to compare the rate of change in *NR3C1*- and *NR3C2*-Thickness Similarity during the first few decades (increasing similarity) and past few decades (decreasing similarity) of the lifespan. The *NR3C1*-Thickness Similarity had a steeper age-related increase in participants < 28 years (mean_difference_ = 2.34e-3, *P* < 0.001). Thus, changes in cortical thickness appeared to be more strongly associated with the distribution of *NR3C1* early in life. Conversely, participants > 68 years displayed no difference in the rate of age-related decline in *NR3C1*- and *NR3C2*-Thickness Similarity (mean_difference_ = 8.98e-4, *P* = 6.41e-2).

### Lifespan Variation in *NR3C1* and *NR3C2* Expression

**Figure 3:**
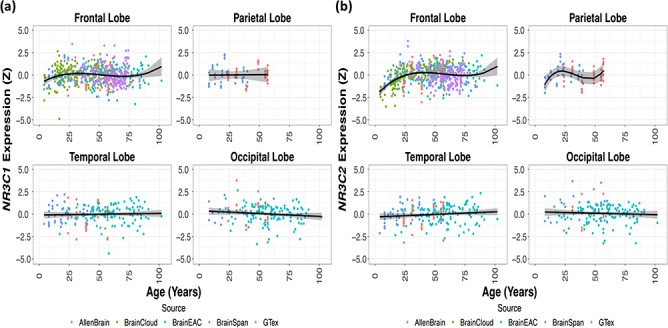
Age-related variation in *NR3C1* and *NR3C2* mRNA expression from 5 pooled sources of human post-mortem gene expression databases (plotted with a cubic smoother). (*a*) Expression of *NR3C1* mRNA in the 4 cerebral lobes. Age did not predict *NR3C1* expression in any of the cortical lobes (Frontal: *F*_(3,493)_ = 3.64, *P* = 0.15; Parietal: *F*_(1,19)_ = 0.01, *P* = 0.95; Occipital: *F*_(1,146)_ = 2.32, *P* = 0.78; Temporal: *F*_(1,139)_ = 0.21, *P* = 0.95). Models with linear age terms fit all regions except the frontal lobe, which had a cubic age term. (*b*) Expression of *NR3C2* mRNA in the 4 cerebral lobes. Age did not predict *NR3C2* expression in the Parietal (*F*_(3,19)_ = 2.16, *P*_FDR_ = 2.80e-01), Occipital (*F*_(1,146)_ = 0.51, *P* = 6.46e-01), or Temporal (*F*_(1,139)_ = 2.41, *P* = 2.80e-01) lobes. A cubic age term did predict *NR3C2* expression in the Frontal lobe (*F*_(3,493)_ = 25.74, *P* = 2.00e-14).

A total of 502 donor brains (66.93% male) were included in this analysis. Donor age ranged from 4 to 102 years (mean = 47.25, SD = 20.19). Lobar *NR3C1* expression did not change with age (Fig. 3*a*). For *NR3C2*, only the frontal lobe displayed age-related increases in expression, which appear to be confined to the early years (Fig. 3*b*).

Additional analysis of age-related changes in gene expression was conducted for each sampled region of the 5 databases (Supplementary Tables S8 and S9). None of the cortical regions sampled displayed age-related changes in *NR3C1* expression. Expression of *NR3C2* increased with age in 5 sampled regions and decreased with age in 2 regions.

### Co-expression of *NR3C1* and *NR3C2* with Cell-Specific Genes

Both *NR3C1* and *NR3C2* were co-expressed negatively with the majority of genes specific to astrocytes (74% and 72%, respectively), CA1 pyramidal cells (72% for both), and microglia (69% and 71%, respectively). Of the 3 cell types, CA1 pyramidal genes had the strongest negative co-expression with both *NR3C1* and *NR3C2* (Table 2).

**Table 2:** Inter-regional co-expression of *NR3C1* and *NR3C2* with cell-specific genes

**Cell type**	**Genes (n)**	***NR3C1***			***NR3C2***		
		**Avg. r**	**p**	**Corrected p (FDR)**	**Avg. r**	**p**	**Corrected p (FDR)**
Astrocyte	54	−0.34	2.00E-04	9.00E-04	−0.34	2.00E-04	9.00E-04
CA1 pyramidal	103	−0.26	1.00E-04	9.00E-04	−0.28	1.00E-04	9.00E-04
Endothelial	57	0.11	1.97E-01	2.53E-01	0.09	2.98E-01	3.83E-01
Ependymal	84	−0.11	1.33E-01	1.99E-01	−0.08	2.36E-01	3.54E-01
Interneuron	100	0.01	8.53E-01	8.53E-01	−0.02	8.29E-01	8.29E-01
Microglia	48	−0.27	5.70E-03	1.71E-02	−0.28	2.90E-03	8.70E-03
Mural	25	−0.11	4.13E-01	4.65E-01	−0.12	3.57E-01	4.02E-01
Oligodendrocyte	60	0.15	6.92E-02	1.25E-01	0.11	1.74E-01	3.13E-01
S1 Pyramidal	73	0.17	3.32E-02	7.47E-02	0.11	1.55E-01	3.13E-01

Avg.r, average correlation coefficient; FDR, false discovery rate corrected.

### Gene-Thickness Similarities that Increase with Age in Late Life

In older participants (age > 68) both *NR3C1*- and *NR3C2*-Thickness Similarities decreased with age. Other biological processes (non-corticosteroid mediated) may be involved. Therefore, we tested for gene-thickness similarities that increase with age in older participants. Of the 2511 genes with consistent cortical profiles, 455 exhibited an increased gene-thickness similarity with age. After conducting a GO enrichment analysis on these 455 genes, no GO terms survived FDR correction. The top 10 GO terms can be found in Supplementary Table S10.

Finally, we compared the *NR3C1-* and *NR3C2-*Thickness Similarities with those calculated for all other genes that pass the 2-stage consistency filtering (*n*_other_ = 2509). To do so, we generated individual-level gene-thickness profile similarities for all 2511 genes (2509 plus *NR3C1* and *NR3C2)*. Next we calculated average gene-thickness profile similarities for each gene within 3 age groups: young (age <27 years; *n* = 8080), middle-aged (age 27–60 years; *n* = 9171), and elderly (age >60 years; *n* = 12 892). The distribution of the 2511 values of the average gene-thickness profile similarities can be seen in Figure S6. Among the negatively correlated genes, *NR3C1* ranked 140, 25, and 44 for the young, middle-aged, and elderly groups, respectively (the top 12%; Fig. S6, dotted line). The *NR3C2-*Thickness Similarity ranked 1, 1, and 7 for the young, middle-aged, and elderly groups, respectively (the top 1%; Fig. S6, solid line).

## Discussion

With a large sample of 21 251 participants, we investigated the relationship between the inter-regional profile of cortical thickness and the inter-regional profile of corticosteroid receptor gene (*NR3C1* and *NR3C2*) expression across the lifespan. In all 34 cortical regions, cortical thickness was associated strongly with age. This is consistent with previous lifespan studies (Salat et al. 2004). Additionally, we show that the cortical profile of *NR3C1* and *NR3C2* mRNA expression was negatively correlated with the profile of cortical thickness. Previous human studies report associations between glucocorticoid exposure and thinner cortex (Kremen et al. 2010; Davis et al. 2013). Moreover, remodeling of dendritic and synaptic architecture is involved in both typical aging (Huttenlocher 1979; Nakamura et al. 1985; de Brabander et al. 1998) and glucocorticoid-associated (Liston and Gan 2011) cortical thinning. Although we cannot rule out the involvement of mineralocorticoids in shaping cortical thickness, this is unlikely for several reasons. Compared with glucocorticoids, mineralocorticoids have much lower plasma levels and more restricted passage across the blood brain barrier; for these reasons, glucocorticoids outcompete mineralocorticoids in their binding to GR and MR in brain tissue (Reul and De Kloet 1985; Yongue and Roy 1987; Arriza et al. 1988; Rupprecht et al. 1993; Geerling and Loewy 2009). Here we present some evidence that the overlapping mechanisms of age- and glucocorticoid-related cortical thinning may be mediated by the 2 corticosteroid receptors.

The *NR3C1*-Thickness Similarity was on average stronger in males than in females. This is consistent with our previous study in which the inter-regional profile of age-related decreases in cortical thickness showed a nominally stronger similarity in male (*R*^2^ = 0.46) versus female (*R*^2^ = 0.30) adolescents (Wong et al. 2017). It is of note that, in the same study, the strongest decreases in cortical thickness were found in cortical regions with high expression of both *AR* and *NR3C1* genes. This synergistic action of *AR* and *NR3C1* might be absent in case of *NR3C2*. This may contribute to the opposite pattern of sex differences in the case of the *NR3C2*-Thickness Similarity (Female>Male).

Our co-expression investigation of cell-specific genes and *NR3C1*/*NR3C2* help explain mechanisms involved in glucocorticoid-associated cortical thinning. The majority of genes associated with CA1 pyramidal cells, microglia, and astrocytes are co-expressed negatively with both *NR3C1*/*NR3C2*. Our previous study of adolescents used a similar technique and identified the same 3 cell types as associated with cortical thickness and thinning (Shin et al. 2017). The association of genes specific to CA1 pyramidal cells with *NR3C1* and *NR3C2* supports the potential role of reduced dendritic complexity in age- and glucocorticoid-related variations in cortical thickness. Areas with greater dendritic complexity will likely require more astrocyte support; dexamethasone, a synthetic glucocorticoid, has been shown to block differentiation of astrocytes (Sabolek et al. 2006). Activation of microglia is also negatively regulated by glucocorticoids (Rivest 2009). The potential reduction in astrocyte density and inactivation of microglia in regions with high *NR3C1* and *NR3C2* expression may contribute to the observed thinner cortex in these regions.

In the first few decades of life, a steeper increase in *NR3C1*-Thickness Similarity, compared with *NR3C2*, suggests the involvement of glucocorticoid-associated GR activation in shaping cortical maturation. Similarly, our previous work, in an adolescent sample, revealed a negative association between *NR3C1* mRNA expression and age-related decreases in cortical thickness (Parker et al. 2017; Wong et al. 2017). Therefore, stress may potentially play a role in the typical cortical thinning observed in early life. From childhood through to early adulthood, there is a combination of a rapid decline in cortical thickness (Gogtay et al. 2004; Raznahan et al. 2010; Tamnes et al. 2010) concurrent with a unique blend of novel stress exposures and increases in stress- and sex-hormone production (Lupien et al. 2009; Eiland and Romeo 2013). The increased glucocorticoid exposure may produce GR-mediated reductions in neuronal excitation, dendritic and synaptic architecture, and, to a lesser extent, cellular apoptosis (Almeida et al. 2000; Crochemore et al. 2005; Radley 2005; Cerqueira et al. 2007; Prager and Johnson 2009). On the other hand, the constitutively occupied MR has been shown to be neuroprotective, to be anti-apoptotic, and to increase neuronal excitability (Almeida et al. 2000; Crochemore et al. 2005; Prager and Johnson 2009). With the transcription rate of the MR less than that of GR (Arriza et al. 1988; Rupprecht et al. 1993), the net response may lead to a relative reduction in dendritic and synaptic complexity. Alterations in synapse and dendritic spine formation have been associated with circadian peaks in circulating glucocorticoids (potential GR role); conversely, maintenance of those structural-alterations occurred during troughs/low levels of circulating glucocorticoids (potential MR role; Liston and Gan 2011; Liston et al. 2013). In addition, an MR:GR balance is suggested to be critical for healthy neuronal activity (Sousa et al. 2008; Oitzl et al. 2010); this may extend to shaping and maintaining age-related changes in cortical thickness.

Glucocorticoid exposure may play a role in shaping brain maturation but brain aging may be governed by different biological processes. In midlife, we observed little change in *NR3C1*- or *NR3C2*-Thickness Similarity. There are several potential reasons: by midlife, 1) there may be adequate stressor coping and maintenance of an MR:GR balance; 2) a ceiling effect may occur, limiting the degree of similarity between cortical thickness and *NR3C1*/*NR3C2* distribution; or 3) competing biological processes may interfere with age-related increases in *NR3C1*- and *NR3C2*-Thickness Similarity. In the seventh decade, the maintenance of a strong *NR3C1*- or *NR3C2*-Thickness Similarity begins to decline. That is, the inter-regional profile of cortical thickness no longer resembles the distribution of the GRs mRNA expression as tightly as during midlife. Dysregulation of the HPA axis and a decrease in sensitivity to glucocorticoids are evident during aging (Sapolsky et al. 1986; Ferrari et al. 2001; Seeman et al. 2001). This may disrupt the midlife-maintenance mechanisms mentioned above (reason 1). In a study of stress-associated gene expression, young rats exposed to the same thermic stressor as older rats exhibited differential gene expression. Specifically, older rats had a less induction of stress-response genes and an increase in oxidative stress-associated genes (Zhang et al. 2002). Oxidative stress, denoted by the cellular accumulation of free-radical by-products, is closely linked to aging (Lee et al. 2000; Terman and Brunk 2006). Moreover, neuro-inflammation is strongly associated with aging (Lee et al. 2000). These are 2 late life biological processes that may alter the strength of association between the profile of cortical thickness and the profile of *NR3C1* (or *NR3C2*). Therefore, senescence is linked to disruption of many biological processes, beyond the dysregulation of the HPA axis, all of which may impact the patterned changes in cortical thickness.

Here we used gene expression data, sampled from different regions of the human cerebral cortex and deposited in 5 separate databases with over 500 post-mortem donor brains, to show that the expression of *NR3C1* and, with a few exceptions, also of *NR3C2* do not change with age. This rules out the potential confounding effect of age-related changes in gene expression driving observed variations in *NR3C1*- or *NR3C2*-Thickness Similarity with age. Previous studies have shown an increase in *NR3C1* mRNA expression in cerebral cortex with age (Perlman et al. 2007; Sinclair et al. 2011). Our results may vary due to differences in sample size, age range, and brain regions sampled. Additionally, unlike previous studies, age was modeled as continuous variable in our analysis. Also, increase in *NR3C1* and/or *NR3C2* expression with age would likely result in a lifespan strengthening of both the *NR3C1*- and *NR3C2*-Thickness Similarity, which was not observed.

We acknowledge that there are various methods used for mapping gene expression data to a brain atlas space. Arnatkevic̆iūtė et al. (2019) have reviewed a number of approaches (including the approach employed here) used for relating gene expression data from the Allen Human Brain Atlas to various neuroimaging datasets. As pointed out by Arnatkevic̆iūtė et al. (2019), while valid, our original approach did not include probes unassigned to an annotated gene and did not filter probe expression based on their intensity. On the other hand, we applied strict consistency criteria (profile similarity across donors and atlases), which have minimized possible technical sources of noise in the expression data.

## Conclusions

The aim of this study was to determine the association between corticosteroids and cortical thickness across the lifespan. This was accomplished by using a large sample of participants in conjunction with human post-mortem gene expression data from several publicly available databases. The 2 corticosteroid receptors may be involved in shaping the profile of cortical thickness early in the lifespan. In midlife, there is a relative maintenance of a strong association between cortical thickness and corticosteroid receptor expression across the cerebral cortex. In late life, this association deteriorates. The results suggest that corticosteroids may influence brain structure to a varying degree throughout life and, as such, may contribute to age- and stress-related variations in cognitive functioning and mental health.

## Notes

SYS: The Canadian Institutes of Health Research and the Heart and Stroke Foundation of Canada fund the SYS. Computations were performed on the GPC supercomputer at the SciNet HPC Consortium. SciNet is funded by the Canada Foundation for Innovation under the auspices of Compute Canada, the Government of Ontario, Ontario Research Fund—Research Excellence, and the University of Toronto.

The Study of Health in Pomerania (SHIP) is part of the Community Medicine Research net (http://www.medizin.uni-greifswald.de/icm) of the University Medicine Greifswald, which is supported by the German Federal State of Mecklenburg-West Pomerania. MRI scans in SHIP and SHIP-TREND have been supported by a joint grant from Siemens Healthineers, Erlangen, Germany and the Federal State of Mecklenburg-West Pomerania. This study was further supported by the German Centre of Neurodegenerative Diseases and the EU-JPND Funding for BRIDGET (FKZ: 01ED1615). LBC1936: The Lothian Birth Cohort 1936 (LBC1936) is supported by Age UK (Disconnected Mind project) and by the UK Medical Research Council (MRC; G0701120, G1001245, MR/M013111/1). OASIS: Data were provided (in part) by OASIS-3: Principal Investigators: T. Benzinger, D. Marcus, J. Morris; NIH P50AG00561, P30NS09857781, P01AG026276, P01AG003991, R01AG043434, UL1TR000448, R01EB009352. HCP: Data were provided (in part) by the Human Connectome Project, WU-Minn Consortium (Principal Investigators: David Van Essen and Kamil Ugurbil; 1U54MH091657) funded by the 16 NIH Institutes and Centers that support the NIH Blueprint for Neuroscience Research; and by the McDonnell Center for Systems Neuroscience at Washington University. MAS: The work was supported by an Australian National Health and Medical Research Council Program Grant (ID1093083), and philanthropic funding through The Dementia Momentum. OATS: We acknowledge the contribution of the OATS research team (https://cheba.unsw.edu.au/project/older-australian-twins-study) to this study. The OATS study has been funded by a National Health & Medical Research Council (NHMRC) and Australian Research Council (ARC) Strategic Award Grant of the Ageing Well, Ageing Productively Program (ID No. 401162) and NHMRC Project Grants (ID 1045325 and 1085606). We thank the participants for their time and generosity in contributing to this research. *Conflict of Interest*: None declared.

## Supplementary Material

Supplementary_Material_29_04_2019_bhz108

## References

[ref1] Almeida OFX, Condé GL, Crochemore G, Demeneix BA, Fischer D, Hassan AHS, Meyer M, Holsboer F, Michaelidis TM 2000 Subtle shifts in the ratio between pro- and antiapoptotic molecules after activation of corticosteroid receptors decide neuronal fate. FASEB J. 14:779–790. 10744634 10.1096/fasebj.14.5.779

[ref2] Ardlie KG, DeLuca DS, Segrè AV, Sullivan TJ, Young TR, Gelfand ET, Trowbridge CA, Maller JB, Tukiainen T, Lek M et al. 2015 The Genotype-Tissue Expression (GTEx) pilot analysis: multitissue gene regulation in humans. Science. 348:648–660. 25954001 10.1126/science.1262110 PMC4547484

[ref3] Arloth J, Bogdan R, Weber P, Frishman G, Menke A, Wagner KV, Balsevich G, Schmidt MV, Karbalai N, Czamara D et al. 2015 Genetic differences in the immediate transcriptome response to stress predict risk-related brain function and psychiatric disorders. Neuron. 86:1189–1202. 26050039 10.1016/j.neuron.2015.05.034 PMC4490780

[ref4] Arnatkevic̆iūtė A, Fulcher BD, Fornito A 2019 A practical guide to linking brain-wide gene expression and neuroimaging data. Neuroimage. 189:353–367. 30648605 10.1016/j.neuroimage.2019.01.011

[ref5] Arriza JL, Simerly RB, Swanson LW, Evans RM 1988 The neuronal mineralocorticoid receptor as a mediator of glucocorticoid response. Neuron. 1:887–900. 2856104 10.1016/0896-6273(88)90136-5

[ref6] de Brabander JM, Kramers RJK, Uylings HBM 1998 Layer-specific dendritic regression of pyramidal cells with ageing in the human prefrontal cortex. Eur J Neurosci. 10:1261–1269. 9749780 10.1046/j.1460-9568.1998.00137.x

[ref7] Cerqueira JJ, Taipa R, Uylings HBM, Almeida OFX, Sousa N 2007 Specific configuration of dendritic degeneration in pyramidal neurons of the medial prefrontal cortex induced by differing corticosteroid regimens. Cereb Cortex. 17:1998–2006. 17082516 10.1093/cercor/bhl108

[ref10] Cook SC, Wellman CL 2004 Chronic stress alters dendritic morphology in rat medial prefrontal cortex. J Neurobiol. 60:236–248. 15266654 10.1002/neu.20025

[ref11] Crochemore C, Lu J, Wu Y, Liposits Z, Sousa N, Holsboer F, Almeida OFX 2005 Direct targeting of hippocampal neurons for apoptosis by glucocorticoids is reversible by mineralocorticoid receptor activation. Mol Psychiatry. 10:790–798. 15940303 10.1038/sj.mp.4001679

[ref12] Davis EP, Sandman CA, Buss C, Wing DA, Head K 2013 Fetal glucocorticoid exposure is associated with preadolescent brain development. Biol Psychiatry. 74:647–655. 23611262 10.1016/j.biopsych.2013.03.009 PMC3985475

[ref13] Desikan RS, Segonne F, Fischl B, Quinn BT, Dickerson BC, Blacker D, Buckner RL, Dale AM, Maguire RP, Hyman BT et al. 2006 An automated labeling system for subdividing the human cerebral cortex on MRI scans into gyral based regions of interest. Neuroimage. 31:968–980. 16530430 10.1016/j.neuroimage.2006.01.021

[ref14] Eiland L, Romeo RD 2013 Stress and the developing adolescent brain. Neuroscience. 249:162–171. 23123920 10.1016/j.neuroscience.2012.10.048 PMC3601560

[ref15] Epel ES, Blackburn EH, Lin J, Dhabhar FS, Adler NE, Morrow JD, Cawthon RM 2004 Accelerated telomere shortening in response to life stress. Proc Natl Acad Sci. 101:17312–17315. 15574496 10.1073/pnas.0407162101 PMC534658

[ref16] Ferrari E, Cravello L, Muzzoni B, Casarotti D, Paltro M, Solerte SB, Fioravanti M, Cuzzoni G, Pontiggia B 2001 Age-related changes of the hypothalamic-pituitary-adrenal axis: pathophysiological correlates. Eur J Endocrinol. 144:319–329. 11275940 10.1530/eje.0.1440319

[ref17] Fjell AM, Grydeland H, Krogsrud SK, Amlien I, Rohani DA, Ferschmann L, Storsve AB, Tamnes CK, Sala-Llonch R, Due-Tønnessen P et al. 2015 Development and aging of cortical thickness correspond to genetic organization patterns. Proc Natl Acad Sci. 112:15462–15467. 26575625 10.1073/pnas.1508831112 PMC4687601

[ref18] French L, Paus T 2015 A FreeSurfer view of the cortical transcriptome generated from the Allen Human Brain Atlas. Front Neurosci. 9:1–5. 26441498 10.3389/fnins.2015.00323 PMC4584957

[ref19] Gassen NC, Chrousos GP, Binder EB, Zannas AS 2017 Life stress, glucocorticoid signaling, and the aging epigenome: implications for aging-related diseases. Neurosci Biobehav Rev. 74:356–365. 27343999 10.1016/j.neubiorev.2016.06.003

[ref20] Geerling JC, Loewy AD 2009 Aldosterone in the brain. AJP Ren Physiol. 297:F559–F576. 10.1152/ajprenal.90399.2008 PMC2739715 19261742

[ref21] Gesing A, Bilang-Bleuel A, Droste SK, Linthorst AC, Holsboer F, Reul JM 2001 Psychological stress increases hippocampal mineralocorticoid receptor levels: involvement of corticotropin-releasing hormone. J Neurosci. 21:4822–4829. 11425909 10.1523/JNEUROSCI.21-13-04822.2001 PMC6762361

[ref22] Gogtay N, Giedd JN, Lusk L, Hayashi KM, Greenstein D, Vaituzis AC, Nugent TF 3rd, Herman DH, Clasen LS, Toga AW et al. 2004 Dynamic mapping of human cortical development during childhood through early adulthood. Proc Natl Acad Sci. 101:8174–8179. 15148381 10.1073/pnas.0402680101 PMC419576

[ref23] Haussmann MF, Heidinger BJ 2015 Telomere dynamics may link stress exposure and ageing across generations. Biol Lett. 11:20150396. 26538535 10.1098/rsbl.2015.0396 PMC4685533

[ref25] Huttenlocher PR 1979 Synaptic density in human frontal cortex—developmental changes and effects of aging. Brain Res. 163:195–205. 427544 10.1016/0006-8993(79)90349-4

[ref26] Jaffe AE, Hyde T, Kleinman J, Weinbergern DR, Chenoweth JG, McKay RD, Leek JT, Colantuoni C 2015 Practical impacts of genomic data “cleaning” on biological discovery using surrogate variable analysis. BMC Bioinformatics. 16:1–10. 26545828 10.1186/s12859-015-0808-5 PMC4636836

[ref27] Kolb B, Gibb R, Gorny G 2003 Experience-dependent changes in dendritic arbor and spine density in neocortex vary qualitatively with age and sex. Neurobiol Learn Mem. 79:1–10. 12482673 10.1016/s1074-7427(02)00021-7

[ref28] Koss WA, Belden CE, Hristov AD, Juraska JM 2014 Dendritic remodeling in the adolescent medial prefrontal cortex and the basolateral amygdala of male and female rats. Synapse. 68:61–72. 24105875 10.1002/syn.21716

[ref29] Kremen WS, O’Brien RC, Panizzon MS, Prom-Wormley E, Eaves LJ, Eisen SA, Eyler LT, Hauger RL, Fennema-Notestine C, Fischl B et al. 2010 Salivary cortisol and prefrontal cortical thickness in middle-aged men: a twin study. Neuroimage. 53:1093–1102. 20156572 10.1016/j.neuroimage.2010.02.026 PMC4034538

[ref30] Lee CK, Weindruch R, Prolla TA 2000 Gene-expression profile of the ageing brain in mice. Nat Genet. 25:294–297. 10888876 10.1038/77046

[ref31] Li M, Santpere G, Kawasawa YI, Evgrafov OV, Gulden FO, Pochareddy S, Sunkin SM, Li Z, Shin Y, Zhu Y et al. 2018 Integrative functional genomic analysis of human brain development and neuropsychiatric risks. Science. 362: eaat7615. 10.1126/science.aat7615 PMC6413317 30545854

[ref32] Liston C, Cichon JM, Jeanneteau F, Jia Z, Chao MV, Gan W-B 2013 Circadian glucocorticoid oscillations promote learning- dependent synapse formation and maintenance. Nat Neurosci. 16:698–705. 23624512 10.1038/nn.3387 PMC3896394

[ref33] Liston C, Gan W-B 2011 Glucocorticoids are critical regulators of dendritic spine development and plasticity in vivo. Proc Natl Acad Sci. 108:16074–16079. 21911374 10.1073/pnas.1110444108 PMC3179117

[ref34] Lupien SJ, McEwen BS, Gunnar MR, Heim C 2009 Effects of stress throughout the lifespan on the brain, behaviour and cognition. Nat Rev Neurosci. 10:434–445. 19401723 10.1038/nrn2639

[ref35] Mazziotta J, Toga A, Evans A, Fox P, Lancaster J, Zilles K, Woods R, Paus T, Simpson G, Pike B et al. 2001 A probabilistic atlas and reference system for the human brain: International Consortium for Brain Mapping (ICBM). Philos Trans R Soc B Biol Sci. 356:1293–1322. 10.1098/rstb.2001.0915 PMC1088516 11545704

[ref36] Menke A, Arloth J, Pütz B, Weber P, Klengel T, Mehta D, Gonik M, Rex-Haffner M, Rubel J, Uhr M et al. 2012 Dexamethasone stimulated gene expression in peripheral blood is a sensitive marker for glucocorticoid receptor resistance in depressed patients. Neuropsychopharmacology. 37:1455–1464. 22237309 10.1038/npp.2011.331 PMC3327850

[ref37] Nakamura S, Akiguchi I, Kameyama M, Mizuno N 1985 Age-related changes of pyramidal cell basal dendrites in layers III and V of human motor cortex: a quantitative golgi study. Acta Neuropathol. 65:281–284. 3976364 10.1007/BF00687009

[ref38] Niwa M, Jaaro-Peled H, Tankou S, Seshadri S, Hikida T, Matsumoto Y, Cascella NG, Kano S, Ozaki N, Nabeshima T et al. 2013 Adolescent stress–induced epigenetic control of dopaminergic neurons via glucocorticoids. Science. 339:335–339. 23329051 10.1126/science.1226931 PMC3617477

[ref39] Oitzl MS, Champagne DL, van der Veen R, de Kloet ER 2010 Brain development under stress: hypotheses of glucocorticoid actions revisited. Neurosci Biobehav Rev. 34:853–866. 19631685 10.1016/j.neubiorev.2009.07.006

[ref40] Parker N, Wong AP, Leonard G, Perron M, Pike B, Richer L, Veillette S, Pausova Z, Paus T 2017 Income inequality, gene expression, and brain maturation during adolescence. Sci Rep. 7:1–11. 28784996 10.1038/s41598-017-07735-2 PMC5547165

[ref41] Perlman WR, Webster MJ, Herman MM, Kleinman JE, Weickert CS 2007 Age-related differences in glucocorticoid receptor mRNA levels in the human brain. Neurobiol Aging. 28:447–458. 16540204 10.1016/j.neurobiolaging.2006.01.010

[ref42] Prager EM, Johnson LR 2009 Stress at the synapse: signal transduction mechanisms of adrenal steroids at neuronal membranes. Sci Signal. 2. 10.1126/scisignal.286re5 19724063

[ref43] Radley JJ 2005 Repeated stress induces dendritic spine loss in the rat medial prefrontal cortex. Cereb Cortex. 16:313–320. 15901656 10.1093/cercor/bhi104

[ref44] Raznahan A, Lee Y, Stidd R, Long R, Greenstein D, Clasen L, Addington A, Gogtay N, Rapoport JL, Giedd JN 2010 Longitudinally mapping the influence of sex and androgen signaling on the dynamics of human cortical maturation in adolescence. Proc Natl Acad Sci. 107:16988–16993. 20841422 10.1073/pnas.1006025107 PMC2947865

[ref45] Reul JMHM, De Kloet ER 1985 Two receptor systems for corticosterone in rat-brain - microdistribution and differential occupation. Endocrinology. 117:2505–2511. 2998738 10.1210/endo-117-6-2505

[ref46] Rivest S 2009 Regulation of innate immune response in the brain. Nat Rev Immunol. 9:429–439. 19461673 10.1038/nri2565

[ref47] Rupprecht R, Arriza JL, Spengler D, Reul JM, Evans RM, Holsboer F, Damm K 1993 Transactivation and synergistic properties of the mineralocorticoid receptor: relationship to the glucocorticoid receptor. Mol Endocrinol. 7:597–603. 8388999 10.1210/mend.7.4.8388999

[ref48] Sabolek M, Herborg A, Schwarz J, Storch A 2006 Dexamethasone blocks astroglial differentiation from neural precursor cells. Neuroreport. 17:1719–1723. 17047460 10.1097/01.wnr.0000236862.08834.50

[ref49] Salat DH, Buckner RL, Snyder AZ, Greve DN, Desikan RSR, Busa E, Morris JC, Dale AM, Fischl B 2004 Thinning of the cerebral cortex in aging. Cereb Cortex. 14:721–730. 15054051 10.1093/cercor/bhh032

[ref50] Sapolsky RM 2015 Stress and the brain: individual variability and the inverted-U. Nat Neurosci. 18:1344–1346. 26404708 10.1038/nn.4109

[ref51] Sapolsky RM, Krey LC, McEwen BS 1986 The neuroendocrinology of stress and aging: the glucorticoid cascade hypothesis. Endocr Rev. 7:284–301. 3527687 10.1210/edrv-7-3-284

[ref52] Seeman TE, Singer B, Wilkinson CW, McEwen B 2001 Gender differences in age-related changes in HPA axis reactivity. Psychoneuroendocrinology. 26:225–240. 11166486 10.1016/s0306-4530(00)00043-3

[ref53] Selemon LD 2013 A role for synaptic plasticity in the adolescent development of executive function. Transl Psychiatry. 3:e238–e239. 23462989 10.1038/tp.2013.7 PMC3625918

[ref54] Shalev I, Moffitt T, Sugden K, Williams B, Houts R, Danese A, Mill J, Arseneault L, Caspi A 2013 Exposure to violence during childhood is associated with telomere erosion from 5 to 10 years of age: a longitudinal study. Mol Psychiatry. 18:576–581. 22525489 10.1038/mp.2012.32 PMC3616159

[ref55] Shin J, French L, Xu T, Leonard G, Perron M, Pike GB, Richer L, Veillette S, Pausova Z, Paus T 2017 Cell-specific gene-expression profiles and cortical thickness in the human brain. Cereb Cortex. 28:3267–3277. 10.1093/cercor/bhx197 PMC13017599 28968835

[ref56] Sinclair D, Webster MJ, Wong J, Weickert CS 2011 Dynamic molecular and anatomical changes in the glucocorticoid receptor in human cortical development. Mol Psychiatry. 16:504–515. 20308989 10.1038/mp.2010.28

[ref57] Sousa N, Cerqueira JJ, Almeida OFX 2008 Corticosteroid receptors and neuroplasticity. Brain Res Rev. 57:561–570. 17692926 10.1016/j.brainresrev.2007.06.007

[ref58] Sowell ER, Peterson BS, Kan E, Woods RP, Yoshii J, Bansal R, Xu D, Zhu H, Thompson PM, Toga AW 2007 Sex differences in cortical thickness mapped in 176 healthy individuals between 7 and 87 years of age. Cereb Cortex. 17:1550–1560. 16945978 10.1093/cercor/bhl066 PMC2329809

[ref59] Storsve AB, Fjell AM, Tamnes CK, Westlye LT, Overbye K, Aasland HW, Walhovd KB 2014 Differential longitudinal changes in cortical thickness, surface area and volume across the adult life span: regions of accelerating and decelerating change. J Neurosci. 34:8488–8498. 24948804 10.1523/JNEUROSCI.0391-14.2014 PMC6608217

[ref60] Tamnes CK, Østby Y, Fjell AM, Westlye LT, Due-Tønnessen P, Walhovd KB 2010 Brain maturation in adolescence and young adulthood: regional age-related changes in cortical thickness and white matter volume and microstructure. Cereb Cortex. 20:534–548. 19520764 10.1093/cercor/bhp118

[ref61] Terman A, Brunk UT 2006 Oxidative stress accumulation of biological “garbage”, and aging. Antioxid Redox Signal. 8:197–204. 16487053 10.1089/ars.2006.8.197

[ref62] Trabzuni D, Ryten M, Walker R, Smith C, Imran S, Ramasamy A, Weale ME, Hardy J 2011 Quality control parameters on a large dataset of regionally dissected human control brains for whole genome expression studies. J Neurochem. 119:275–282. 21848658 10.1111/j.1471-4159.2011.07432.x PMC3664422

[ref63] Vandekar SN, Shinohara RT, Raznahan A, Roalf DR, Ross M, DeLeo N, Ruparel K, Verma R, Wolf DH, Gur RC et al. 2015 Topologically dissociable patterns of development of the human cerebral cortex. J Neurosci. 35:599–609. 25589754 10.1523/JNEUROSCI.3628-14.2015 PMC4293413

[ref64] Vyas A, Mitra R, Rao BSS, Chattarji S 2002 Chronic stress induces contrasting patterns of dendritic remodeling in hippocampal and amygdaloid neurons. J Neurosci. 22:6810–6818. 12151561 10.1523/JNEUROSCI.22-15-06810.2002 PMC6758130

[ref65] Wong A, French L, Leonard G, Perron M, Pike GB, Richer L, Veillette S, Pausova Z, Paus T 2017 Inter-regional variations in gene expression and age-related cortical thinning in the adolescent brain. Cereb Cortex. 28:1272–1281. 10.1093/cercor/bhx040 PMC6093352 28334178

[ref66] Wood SN 2006 Generalized additive models: an introduction with R. 1st ed. New York: Taylor & Francis Group.

[ref67] Yongue BG, Roy EJ 1987 Endogenous aldosterone and corticosterone in brain cell nuclei of adrenal-intact rats: regional distribution and effects of physiological variations in serum steroids. Brain Res. 436:49–61. 3690353 10.1016/0006-8993(87)91555-1

[ref68] Zannas AS, Chrousos GP 2017 Epigenetic programming by stress and glucocorticoids along the human lifespan. Mol Psychiatry. 22:640–646. 28289275 10.1038/mp.2017.35

[ref69] Zeisel A, Muñoz-Manchado AB, Codeluppi S, Lönnerberg P, La MG, Juréus A, Marques S, Munguba H, He L, Betsholtz C et al. 2015 Cell types in the mouse cortex and hippocampus revealed by single-cell RNA-seq. Science. 347:1138–1142. 25700174 10.1126/science.aaa1934

[ref70] Zhang HJ, Drake VJ, Morrison JP, Oberley LW, Kregel KC 2002 Molecular biology of thermoregulation selected contribution: differential expression of stress-related genes with aging and hyperthermia. J Appl Physiol. 92:1762–1769. 11896047 10.1152/japplphysiol.00733.2001

