# Supplementary material for: Corticosteroids and Regional Variations in Thickness of the Human Cerebral Cortex across the Lifespan
Source: Cereb Cortex. 2019 Jun 26;30(2):575–86. doi: 10.1093/cercor/bhz108 (PMC7444740; doi:10.1093/cercor/bhz108)
Supplement: Supplementary_Material_29_04_2019_bhz108 [file supplementary_material_29_04_2019_bhz108.docx]

**Table S1. Cohort Imaging Characteristics**

| **Study** | **Study Design** | **FreeSurfer Version** | **MRI Machine Make** | **Field**  **Strength** | **MRI Acquisition Protocol** |
| --- | --- | --- | --- | --- | --- |
| HRC | Longitudinal Population-Based | V5.3 | GE | 1.5T (x2) | T1W, TR=10.916 ms, TE=4.2 ms, slice thickness=1.2 mm, flip angle=15°, matrix size=2563192, FOV=245 mm, max=156 slices) |
| SYS Adolescents | Cross-Sectional  Family-Based | V5.3 | Philips | 1T | T1W, 1-mm, isotropic resolution images acquired with a 3D fast radio frequency (RF)-spoiled gradient-echo scan. |
| IMAGEN | Longitudinal Population-Based | V5.3 | Siemens, Philips, GE, Bruker | 3T (x8) | TR=2,300 ms, TE=2.8 ms, flip angle=9^o^; resolution: 1x1x1 mm^3^ |
| BIL&GIN | Cross-Sectional  Population-Based | V5.3 | Philips | 3T | The acquisition protocol included a high resolution 3D T1-weighted sequence (3D-FFE-TFE; TR = 20 ms; TE = 4.6 ms; flip angle = 10°; inversion time = 800 ms; turbo field echo factor = 65; sense factor = 2; matrix size = 256 × 256 × 180 mm3; 1mm3 isotropic voxel size). |
| LCBC | Longitudinal  Population-Based | V6.0 | Siemens | 1.5 (x2) & 3T | For **1.5T:** Two identical MPRAGE sequences with the following parameters: TR/TE/TI = 2400 ms/3.61 ms/1000 ms, FA = 8°, acquisition matrix = 192 × 192, FOV = 192, 160 sagittal slices with voxel size = 1.25×1.25×1.2 mm; 12-channel coil.  For **3.0T:**  MPRAGE collected using a 24-channel coil. Paramters: 176 sagittally oriented slices obtained using a turbo field echo pulse sequence (TR/TE/TI = 2300 ms/2.98 ms/ 850 ms, FA = 8⁰, acquisition matrix = 256 × 256 with voxel size = 1×1×1 mm). |
| HCP | Cross-Sectional  Family Based | V5.3 | Siemens | 3T | 3D-MPRAGE with 2400 TR, 2.14 TE, flip angle of 8^o^ and a 0.7mm isotropic volxel size. |
| SYS Parents | Cross-Sectional  Family-Based | V5.3 | Siemens | 1.5T | Three-dimensional (3D) radio frequency (RF)-spoiled gradient-echo scan with 140 –160 slices, an isotropic resolution of 1 mm, a repetition time (TR) of 25 ms, an echo time (TE) of 5 ms, and flip angle of 30° |
| DLBS | Cross-Sectional | V5.3 | Philips | 3T | T1-MPRAGE with 160 slices and a repetition time of 8.14 ms. |
| RS | Longitudinal Population-Based | V6.0 | GE Signa | 1.5T | Structural imaging is performed with T1-weighted (T1w), proton density-weighted (PDw) and fluid-attenuated inversion recovery (FLAIR) sequences. The combination of different MR contrasts provided by these sequences can be used for automated brain tissue and white matter lesion segmentation (see section on processing). For this purpose, the T1w scan is acquired in 3D at high in-plane resolution and with thin slices (voxel size). |
| SALD | Cross-Sectional  Population-Based | V5.3 | Siemens | 3T | T1-MPRAGE with 1mm isotropic voxels, a repetition time (TR) of 1900 ms, an echo time (TE) of 2.52 ms, and flip angle of 9° |
| MAS | Cross-Sectional  Population-Based | V5.3 | Philips | 3T | TR = 6.39 ms, TE = 2.9 ms, flip angle = 8◦, matrix size = 256 × 256, FOV = 256 × 256 × 190, and slice thickness = 1 mm with no gap in between, yielding 1 × 1 × 1 mm3 isotropic voxels. |
| FHS | Cross-Sectional  Population Based | V5.3 | Siemens | 1.5T | Subjects were imaged on a Siemens Magnetom(Munich, Germany), using a T2-weighted double spin-echo coronal imaging sequence of 4mm contiguous slices from nasion to occiput with a repetition time (TR) of 2420 ms, echo time (TE) of TE1 20/TE2 90 ms; echo train length 8 ms; field of view (FOV) 22 cm and an acquisition matrix of 182 × 256 interpolated to a 256 × 256 with one excitation. |
| ASPS_Fam | Cross-Sectional  Family-Based | V5.1.0 | Siemens | 3T | T1-MPRAGE 1x1x1mm |
| OATS | Cross-Sectional  Twin Study | V5.3 | GE  Philips | 1.5T  3T | GE: resolution= 1×1 mm, slice thickness= 1.5 mm, slice number=144, TR (Repetition time) =1530 ms, TE (Echo time) = 3.24 ms, TI (Inversion time)=780 ms, and flip angle=8  Philips: TR/TE = 6.39/2.9 ms, in-plane resolution= 1×1 mm, slice thickness= 1 mm, slice number=190, resulting isotropic voxels of 1×1×1 mm3 |
| OASIS | Longitudinal | V5.3 | Siemens | 3T (x3) | T1-MPRAGE 1x1x1.25mm with a repetition time (TR) of 9.7ms, an echo time (TE) of 4ms, and flip angle of 10° |
| 3C_Dijon | Cross-Sectional | V5.3 | Siemens | 1.5T | T1w using a 3-dimensional inversion recovery fast spoiled-gradient echo sequence (TR = 97 ms; TE = 4 ms; TI = 600 ms; coronal acquisition) |
| LBC1936 | Cross-Sectional  Birth Cohort | V5.1.0 | GE Signa | 1.5T | T1W, coronal FSPGR, TR=10 ms, TE=4 ms, slice thickness=1.3 mm, flip angle=8°, matrix size=192×192, FOV=256×256 mm, max=160 slices. |
| SHIP&TREND | Cross-Sectional  Population Based | V5.3 | Siemens | 1.5T | T1W, TR=1900 ms, TE=3.4 ms, slice thickness=1 mm, flip angle=15°, resolution 1.0x1.0x1.0mm3. |
| HRC: Brazilian High Risk Cohort for Psychiatric Disorders, SYS: Saguenay Youth Study, BIL&GIN: Brain Imaging of Lateralization by the Groupe d'Imagerie Fonctionnelle, LCBC: Lifespan Changes in Brain and Cognition, HCP: Human Connectome Project, SHIP: Study of Health in Pomerania and SHIP-TREND, DLBS: Dallas Lifespan Brain Study, RS: Rotterdam Study, SALD: Southwest University Adult Lifespan Dataset, MAS: Memory and Aging Study, FHS: Framingham Heart Study, ASPS_Fam: Austrian Stroke Prevention Study Family Cohort, OATS: Older Australian Twin Study, OASIS: Open Access Series of Imaging Studies, 3C_Dijon: The 3 City Study - Dijon location, LB1936C: Lothian Birth Cohort 1936. | | | | | |

**Table S2. Description of Gene Expression Databases**

| **Database** | **Description and Data Acquisition** |
| --- | --- |
| Allen Human Brain Atlas | Extensive sampling of cortical and sub-cortical brain structures was carried out using anatomical landmarks identified by histology (Hawrylycz et al. 2012) Gene expression was analyzed using Agilent microarrays. There were a total of six left hemispheres assayed. The age range of the six donors was 24-57 years with only one female donor. |
| BrainCloud | This database included a lifespan analysis of expression patterns in the dorsolateral prefrontal cortex (Colantuoni et al. 2012). Gene expression was measured using microarrays from the National Human Genome Research Institute. The total dataset includes 270 donors of both pre- and post-natal brains. Of those, a total of 206 donors (older than 4 years of age) were included in this analysis. These donors ranged in age from 4 to 78 years of age; 141 (68.45%) donors were male. |
| BrainEAC | This database included samples from 12 regions of 134 donor brains ([www.braineac.org)](http://www.braineac.org)). Of the 12 sampled regions, 3 regions were located in the cerebral cortex (prefrontal, transverse/middle temporal, and visual; (Trabzuni et al. 2011). Gene expression was measured using Affymetrix exon arrays. The 134 donors ranged in age from 16 to 102 years of age; 99 (73.88%) donors were male. |
| BrainSpan | This atlas of the developing brain includes pre- and post-natal donors ([www.brainspan.org)](http://www.brainspan.org)). Here we used the RNA sequencing gene expression data from 11 cortical regions (list of regions can be seen in Supplementary Table 5&6). There were a total of 14 donors included with an age range of 4 to 40 years of age; 8 (57.14%) donors were male. |
| GTEx | This database sampled 53 regions of the brain and body for gene expression ([www.gtexportal.org)](http://www.gtexportal.org)). Data from two cortical regions were included in this analysis (anterior cingulate gyrus and frontal-BA9). Gene expression was measured using RNA sequencing.(Ardlie et al. 2015) Expression data was downloaded using the recount2 resource (<https://jhubiostatistics.shinyapps.io/recount/)> (Collado-Torres et al. 2017). Donor demographics was acquired from the GTEx portal ([www.gtexportal.org)](http://www.gtexportal.org)). Donor age was reported in 10-year bins ranging from 20 to 80. Each donor was assigned the median age of their respective age bin. To introduce variability, age was jittered within the bin range for each donor. A total of 142 donors were included, 99 (69.72%) of which were male. |

**Table S3: Regional Expression of *NR3C1* and *NR3C2***

| Region | *NR3C1* Expression | *NR3C2* Expression |
| --- | --- | --- |
| Banks Superior Temporal Sulcus | 9.501 | 5.938 |
| Caudal Anterior Cingulate | 9.152 | 5.696 |
| Caudal Middle Frontal | 9.500 | 5.917 |
| Cuneus | 9.707 | 6.433 |
| Entorhinal | 8.865 | 5.542 |
| Frontal Pole | 9.357 | 5.696 |
| Fusiform | 9.156 | 5.908 |
| Inferior Parietal | 9.543 | 6.283 |
| Inferior Temporal | 9.216 | 5.930 |
| Insula | 9.244 | 5.780 |
| Isthmus Cingulate | 9.565 | 6.025 |
| Lateral Occipital | 9.595 | 6.369 |
| Lateral Orbitofrontal | 9.432 | 5.928 |
| Lingual | 9.782 | 6.414 |
| Medial Orbitofrontal | 9.191 | 5.855 |
| Middle Temporal | 9.311 | 5.954 |
| Paracentral | 9.501 | 6.121 |
| Parahippocampal | 9.157 | 5.816 |
| Pars Opercularis | 9.346 | 5.853 |
| Pars Orbitalis | 9.530 | 5.970 |
| Pars Triangularis | 9.258 | 6.028 |
| Pericalcarine | 9.651 | 6.562 |
| Post Central | 9.509 | 6.140 |
| Posterior Cingulate | 9.364 | 6.012 |
| Pre-Central | 9.441 | 5.965 |
| Pre-Cuneus | 9.554 | 6.198 |
| Rostral Anterior Cingulate | 9.048 | 5.700 |
| Rostral Middle Frontal | 9.416 | 6.018 |
| Superior Frontal | 9.352 | 5.967 |
| Superior Parietal | 9.488 | 6.215 |
| Superior Temporal | 9.280 | 5.886 |
| Supramarginal | 9.432 | 6.030 |
| Temporal Pole | 8.883 | 5.449 |
| Transverse Temporal | 9.531 | 5.959 |

**Table S4. Gene-Thickness Profile Similarity Simulation Procedure**

| **Cohorts Simulated** | **Simulation Procedure** | **Simulation Accuracy Testing** |
| --- | --- | --- |
| **Framingham Heart Study and**  **Rotterdam Study** | Individual-level Gene*-*Thickness Similarities were calculated at each respective study site. Within each sex separately, these values were binned by age into groups of approximately 20 participants. For each age bin, statistical summaries were shared with the first author for pooling with the other cohorts. With these summaries, 20 individual Gene-Thickness Similarities were simulated from a normal distribution derived from their respective bin means and standard deviations. Each simulated value was assigned the median age of the respective bin. From that, we simulated age within each age-bin by adding, to the median age, random errors generated from a uniform distribution using ‘jitter’ function in R within the given range of the age bin. | To test the accuracy of the simulations, the simulation procedure was repeated 1,000 times. The distribution of Gene-Thickness Similarities from each iteration was compared with the observed values using the Kolmogorov-Smirnov test. The simulation with the lowest test statistic was chosen.  Within the observed data, the effect of age on *NR3C1* and *NR3C2*-Thickness Similarity was determined using linear models adjusting for sex. Linear, quadratic, and cubic age-terms were modelled and a likelihood ratio test was used to determine the appropriate age-term. The same model was then applied to simulated data. NOTE: For the Rotterdam Study, observed data included repeated measures (longitudinal design). Therefore, mixed models (adjusting for repeated measures) were used for observed data, while simulated data were treated as cross sectional.  Age coefficients for simulated data were compared using the normal Z as the test statistic see the equation below:  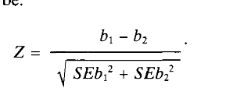 |

**Figure S1**. Framingham Heart Study comparison of observed and simulated data. Similar age trajectories were accomplished with simulation.

**Figure S2**. Rotterdam Study comparison of observed and simulated data. Similar age trajectories were accomplished with simulation.

**Table S5. Comparison of Observed and Simulated *NR3C1*- and *NR3C2*-Thickness Similarity Models**

| ***NR3C1*-Thickness Similarity Comparison** | | | | | |
| --- | --- | --- | --- | --- | --- |
| **Cohort** | **Term**^a^ | **Observed Beta** | **Simulated Beta** | **Z-Test Statistic** | **P** |
| **FHS** | Age  Age^2^ | 0.26  0.57 | 0.47  0.61 | -1.14  -0.25 | 0.25  0.80 |
| **RS** | Age  Age^2^ | 2.69  2.55 | 2.20  2.23 | 2.04  1.43 | 0.04  0.15 |
| ***NR3C2*-Thickness Similarity Comparison** | | | | | |
| **FHS** | Age  Age^2^ | 1.22  0.71 | 1.24  0.75 | -0.12  -0.23 | 0.90  0.82 |
| **RS** | Age  Age^2^ | 4.82  2.89 | 4.26  2.76 | 2.28  0.57 | 0.02  0.57 |
| ^a^ Likelihood ratio tests revealed the quadratic age term fit Framingham and Rotterdam data best for both *NR3C1*- and *NR3C2*-Thickness Similarity.  FHS: Framingham Heart Study, RS: Rotterdam Study  NOTE: For the Rotterdam Study, observed data included repeated measures (longitudinal design). Therefore, mixed models (adjusting for repeated measures) were used for observed data, while simulated data were treated as cross sectional. | | | | | |

**Figure S3**. Cortical thickness by age in each of the 34 cortical regions. Regions are organized in increasing order of *NR3C1* mRNA expression (from top let to bottom right). Mean cortical thickness in each region, represented by the mu symbol, was correlated with *NR3C2* mRNA expression (R^2^=0.83, p= 5.96e-14). Lifespan trajectories are modelled using GAMM analysis with a cubic spline smoother for age (statistics in Supplementary Table 5). The 95% confidence intervals (CI) are shown in dark-grey. R.ACC: rostral anterior cingulate cortex, C. ACC: caudal anterior cingulate cortex, M.: medial, Inf.: inferior, Sup.: superior, P.: posterior, R.: rostral, Mid.: middle, L.: lateral, C.: caudal, BanksSTS: banks of the superior temporal sulcus, Trans.: transverse.

**Table S6. Change in Regional Cortical Thickness with Age**

| **Regions** | **Age** | | **Sex** | |
| --- | --- | --- | --- | --- |
|  | **F_(5, 17534)_** | **p** | **F_(1, 17534)_** | **p** |
| **BanksSTS** | 681.8 | <2.20E-16 | 10.23 | 0.00138 |
| **C.ACC** | 288.5 | <2.20E-16 | 120.1 | <2.20E-16 |
| **C.Mid.Frontal** | 670.6 | <2.20E-16 | 62.76 | 2.48e-15 |
| **Cuneus** | 829.5 | <2.20E-16 | 3.868 | 0.0492 |
| **Entorhinal** | 65.55 | <2.20E-16 | 0.453 | 0.501 |
| **Fusiform** | 441.8 | <2.20E-16 | 18.96 | 1.34e-05 |
| **Inf.Parietal** | 1283 | <2.20E-16 | 127.4 | <2.20E-16 |
| **Inf.Temporal** | 473.9 | <2.20E-16 | 8.385 | 0.00379 |
| **Isthmus Cingulate** | 519.5 | <2.20E-16 | 105.27 | <2.20E-16 |
| **L.Occipital** | 734.4 | <2.20E-16 | 24.48 | 7.57e-07 |
| **L.Orbitofrontal** | 818.7 | <2.20E-16 | 0.159 | 0.69 |
| **Lingual** | 1020 | <2.20E-16 | 29.0 | 7.32e-08 |
| **M.Orbitofrontal** | 704 | <2.20E-16 | 16.61 | 4.61e-05 |
| **Mid.Temporal** | 702.3 | <2.20E-16 | 11.38 | 0.000745 |
| **Parahippocampal** | 86.89 | <2.20E-16 | 75.74 | <2.20E-16 |
| **Paracentral** | 778.5 | <2.20E-16 | 11.73 | 0.000617 |
| **Pars Opercularis** | 868.6 | <2.20E-16 | 3.064 | 0.08 |
| **Pars Orbitalis** | 630.5 | <2.20E-16 | 219.03 | <2.20E-16 |
| **Pars Triangularis** | 978.9 | <2.20E-16 | 45.33 | 1.72e-11 |
| **Pericalcarine** | 463.6 | <2.20E-16 | 0.125 | 0.724 |
| **Post Central** | 834.7 | <2.20E-16 | 96.89 | <2.20E-16 |
| **P.Cingulate** | 795.2 | <2.20E-16 | 32.28 | 1.36e-08 |
| **Precentral** | 640 | <2.20E-16 | 84.07 | <2.20E-16 |
| **Precuneus** | 1377 | <2.20E-16 | 0.902 | 0.342 |
| **R.ACC** | 380.6 | <2.20E-16 | 105.6 | <2.20E-16 |
| **R.Mid.Frontal** | 1088 | <2.20E-16 | 58.69 | 1.94e-14 |
| **Sup.Frontal** | 1147 | <2.20E-16 | 139.3 | <2.20E-16 |
| **Sup.Parietal** | 1042 | <2.20E-16 | 94.02 | <2.20E-16 |
| **Sup.Temporal** | 870.6 | <2.20E-16 | 14.02 | 0.000182 |
| **Supramarginal** | 1127 | <2.20E-16 | 253.9 | <2.20E-16 |
| **Frontal Pole** | 418.9 | <2.20E-16 | 51.34 | 8.07e-13 |
| **Temporal Pole** | 105.9 | <2.20E-16 | 13.57 | 0.000231 |
| **Trans. Temporal** | 503 | <2.20E-16 | 137.9 | <2.20E-16 |
| **Insula** | 674.3 | <2.20E-16 | 62.12 | 3.43e-15 |

**Figure S4.** *NR3C1*- and *NR3C2*-Thickness Similarity lifespan trajectories modelled using GAMM analysis with a cubic spline smoother for age. Data points included with 95% confidence intervals (CI) shown in dark grey. (a) *NR3C1*-Thickness across the lifespan. (b) *NR3C2*-Thickness across the lifespan. (c) *NR3C1*-Thickness Similarity by age modelled in each study separately. (d) *NR3C2*-Thickness Similarity by age modelled in each study separately.

**Table S7. *NR3C1* and *NR3C2*-Thickness Similarity: Age and Sex Effect by Study**

| **Study** | **n** | **Age** | | | **Sex** | | |
| --- | --- | --- | --- | --- | --- | --- | --- |
|  |  | **edf** | **F** | **p** | **df** | **F** | **P** |
| ***NR3C1*-Thickness Similarity** | | | | | | | |
| HRC | 1107 | 2 | 82.74 | <2e-16 | 1 | 0.022 | 0.883 |
| SYS Adolescents | 987 | 3 | 25.63 | 5.51e-16 | 1 | 2.869 | 0.0906 |
| IMAGEN | 1823 | 3 | 225 | <2e-16 | 1 | 14.44 | 0.000148 |
| BIL&GIN | 453 | 5 | 1.272 | 0.292 | 1 | 11.47 | 0.000773 |
| LCBC | 1755 | 6 | 88.8 | <2e-16 | 1 | 0.43 | 0.512 |
| HCP | 1200 | 1 | 8.879 | 0.00317 | 1 | 0.073 | 0.786 |
| SYS Parents | 541 | 1 | 1.002 | 0.317 | 1 | 9.046 | 0.00276 |
| SHIP&TREND | 3021 | 4 | 98.64 | <2e-16 | 1 | 19.88 | 8.56e-06 |
| DLBS | 315 | 1 | 27.97 | 2.27e-07 | 1 | 2.094 | 0.149 |
| RS | 11600 | 6 | 84.18 | <2e-16 | 1 | 8.33 | 0.00391 |
| SALD | 494 | 4 | 0.623 | 0.782 | 1 | 0.015 | 0.901 |
| MAS | 358 | 2 | 0.365 | 0.603 | 1 | 0.863 | 0.353 |
| FHS | 1000 | 4 | 9.323 | 1.19e-07 | 1 | 0.367 | 0.545 |
| ASPS_Fam | 332 | 5 | 3.848 | 0.00154 | 1 | 0.496 | 0.482 |
| OATS | 403 | 2 | 0.143 | 0.705 | 1 | 3.59 | 0.0589 |
| OASIS | 671 | 3 | 3.815 | 0.00486 | 1 | 0.001 | 0.98065 |
| 3C_Dijon | 436 | 1 | 2.173 | 0.141 | 1 | 0.2 | 0.655 |
| LBC1936 | 636 | 1 | 3.901 | 0.0559 | 1 | 2.252 | 0.134 |
| ***NR3C2*-Thickness Similarity** | | | | | | | |
| HRC | 1107 | 1 | 195.5 | <2e-16 | 1 | 0.471 | 0.492 |
| SYS Adolescents | 987 | 3 | 17.7 | 8.74e-11 | 1 | 7.742 | 0.0055 |
| IMAGEN | 1823 | 4 | 73.51 | <2e-16 | 1 | 29.29 | 6.73e-08 |
| BIL&GIN | 453 | 1 | 0.409 | 0.707 | 1 | 22.55 | 2.79e-06 |
| LCBC | 1755 | 6 | 35.38 | <2e-16 | 1 | 6.005 | 0.0143 |
| HCP | 1200 | 1 | 0.214 | 0.644 | 1 | 1.489 | 0.223 |
| SYS Parents | 541 | 1 | 0.19 | 0.663 | 1 | 0.007 | 0.933 |
| SHIP&TREND | 3021 | 4 | 8.808 | 1.37e-08 | 1 | 3.616 | 0.0573 |
| DLBS | 315 | 1 | 1.716 | 0.191 | 1 | 0.113 | 0.737 |
| RS | 11600 | 4 | 195.9 | <2e-16 | 1 | 21.01 | 4.62e-06 |
| SALD | 494 | 5 | 2.838 | 0.0106 | 1 | 1.428 | 0.233 |
| MAS | 358 | 3 | 0.759 | 0.563 | 1 | 0.248 | 0.619 |
| FHS | 1000 | 3 | 27.26 | <2e-16 | 1 | 0.007 | 0.935 |
| ASPS_Fam | 332 | 6 | 3.168 | 0.00582 | 1 | 5.046 | 0.0254 |
| OATS | 403 | 1 | 0.068 | 0.794 | 1 | 0.422 | 0.516535 |
| OASIS | 671 | 3 | 1.816 | 0.106 | 1 | 3.39 | 0.0659 |
| 3C_Dijon | 436 | 1 | 2.029 | 0.155 | 1 | 7.489 | 0.00646 |
| LBC1936 | 636 | 2 | 2.689 | 0.0567 | 1 | 0.196 | 0.658 |
| Edf: estimated degrees of freedom (rounded), df: degrees of freedom. | | | | | | | |

**Figure S5**. Derivative plots for the GAMM lifespan trajectories of *NR3C1*- and *NR3C2*-Thickness Similarities.

**Table S8. *NR3C1* mRNA Expression: Source-Specific Results**

| **Regions** | **Intercept** | | | **Age** | | | **Sex** | | |
| --- | --- | --- | --- | --- | --- | --- | --- | --- | --- |
|  | **beta** | **t** | **p** | **beta** | **t** | **p** | **beta** | **t** | **p** |
| **Allen Human Brain Atlas** | | | | | | | | | |
| **BanksSTS** | 1.47E+00 | 8.14E-01 | 4.17E-01 | -2.37E-02 | -6.02E-01 | 5.48E-01 | NA | NA | NA |
| **C.ACC** | -2.28E-01 | -1.26E-01 | 9.00E-01 | 3.16E-02 | 9.90E-01 | 3.25E-01 | -1.34E+00 | -1.28E+00 | 2.04E-01 |
| **C.Mid.Frontal** | 4.34E-01 | 2.39E-01 | 8.12E-01 | 2.19E-02 | 6.87E-01 | 4.94E-01 | -1.64E+00 | -1.57E+00 | 1.21E-01 |
| **Cuneus** | 2.89E+00 | 1.59E+00 | 1.14E-01 | -3.39E-02 | -1.07E+00 | 2.90E-01 | -1.74E+00 | -1.67E+00 | 9.87E-02 |
| **Entorhinal** | 1.56E-01 | 8.58E-02 | 9.32E-01 | -1.02E-02 | -3.20E-01 | 7.50E-01 | 3.32E-01 | 3.18E-01 | 7.51E-01 |
| **Frontal Pole** | -1.12E+00 | -4.89E-01 | 6.26E-01 | 3.13E-02 | 5.29E-01 | 5.98E-01 |  | NA | NA |
| **Fusiform** | -6.74E-02 | -3.71E-02 | 9.70E-01 | 3.37E-02 | 1.06E+00 | 2.93E-01 | -1.64E+00 | -1.57E+00 | 1.20E-01 |
| **Inf.Parietal** | 1.49E-01 | 8.22E-02 | 9.35E-01 | 2.51E-02 | 7.87E-01 | 4.34E-01 | -1.46E+00 | -1.40E+00 | 1.66E-01 |
| **Inf.Temporal** | 4.46E-02 | 2.46E-02 | 9.80E-01 | 3.05E-02 | 9.58E-01 | 3.41E-01 | -1.61E+00 | -1.54E+00 | 1.27E-01 |
| **Insula** | -3.97E-01 | -2.19E-01 | 8.27E-01 | 3.38E-02 | 1.06E+00 | 2.91E-01 | -1.25E+00 | -1.20E+00 | 2.35E-01 |
| **Isthmus Cingulate** | -6.44E-01 | -3.55E-01 | 7.24E-01 | 2.55E-02 | 8.02E-01 | 4.25E-01 | -5.30E-01 | -5.07E-01 | 6.13E-01 |
| **L.Occipital** | 4.14E-01 | 2.28E-01 | 8.20E-01 | 8.92E-03 | 2.80E-01 | 7.80E-01 | -9.52E-01 | -9.12E-01 | 3.64E-01 |
| **L.Orbitofrontal** | 2.42E+00 | 1.34E+00 | 1.85E-01 | -3.33E-02 | -1.05E+00 | 2.98E-01 | -1.21E+00 | -1.16E+00 | 2.50E-01 |
| **Lingual** | 2.15E+00 | 1.18E+00 | 2.39E-01 | -2.20E-02 | -6.92E-01 | 4.91E-01 | -1.46E+00 | -1.39E+00 | 1.67E-01 |
| **M.Orbitofrontal** | 2.16E+00 | 1.19E+00 | 2.38E-01 | -1.05E-02 | -3.29E-01 | 7.43E-01 | -2.06E+00 | -1.97E+00 | 5.23E-02 |
| **Mid.Temporal** | -1.91E-01 | -1.05E-01 | 9.17E-01 | 3.23E-02 | 1.01E+00 | 3.14E-01 | -1.42E+00 | -1.36E+00 | 1.79E-01 |
| **P.Cingulate** | 1.66E+00 | 9.13E-01 | 3.64E-01 | -8.92E-04 | -2.80E-02 | 9.78E-01 | -1.94E+00 | -1.86E+00 | 6.63E-02 |
| **Paracentral** | 4.06E-01 | 2.24E-01 | 8.24E-01 | 5.04E-03 | 1.58E-01 | 8.75E-01 | -7.44E-01 | -7.13E-01 | 4.78E-01 |
| **Parahippocampal** | 2.12E+00 | 9.74E-01 | 3.33E-01 | -2.46E-02 | -6.13E-01 | 5.41E-01 | -1.56E+00 | -1.37E+00 | 1.75E-01 |
| **Pars Opercularis** | 1.51E-01 | 8.29E-02 | 9.34E-01 | 2.91E-03 | 9.13E-02 | 9.27E-01 | -3.29E-01 | -3.15E-01 | 7.53E-01 |
| **Pars Orbitalis** | -2.51E+00 | -1.61E+00 | 1.11E-01 | 4.39E-02 | 1.21E+00 | 2.27E-01 | NA | NA | NA |
| **Pars Triangularis** | 5.93E+00 | 2.60E+00 | 1.08E-02 | -6.94E-02 | -1.63E+00 | 1.06E-01 | -1.15E+00 | -1.10E+00 | 2.73E-01 |
| **Pericalcarine** | 1.59E+00 | 8.74E-01 | 3.85E-01 | 4.47E-04 | 1.40E-02 | 9.89E-01 | -1.93E+00 | -1.84E+00 | 6.85E-02 |
| **Post Central** | 7.77E-01 | 4.28E-01 | 6.70E-01 | 1.26E-02 | 3.96E-01 | 6.93E-01 | -1.58E+00 | -1.51E+00 | 1.35E-01 |
| **Precentral** | 1.81E+00 | 9.99E-01 | 3.20E-01 | -4.32E-03 | -1.36E-01 | 8.93E-01 | -1.96E+00 | -1.87E+00 | 6.43E-02 |
| **Precuneus** | 2.70E+00 | 1.49E+00 | 1.41E-01 | -2.99E-02 | -9.38E-01 | 3.51E-01 | -1.71E+00 | -1.64E+00 | 1.05E-01 |
| **R.ACC** | 2.72E-01 | 1.50E-01 | 8.81E-01 | 2.84E-02 | 8.91E-01 | 3.75E-01 | -1.77E+00 | -1.70E+00 | 9.31E-02 |
| **R.Mid.Frontal** | 1.35E+00 | 7.46E-01 | 4.57E-01 | 5.71E-03 | 1.79E-01 | 8.58E-01 | -1.92E+00 | -1.84E+00 | 6.98E-02 |
| **Sup.Frontal** | 8.45E-01 | 4.66E-01 | 6.43E-01 | 1.47E-02 | 4.61E-01 | 6.46E-01 | -1.76E+00 | -1.69E+00 | 9.50E-02 |
| **Sup.Parietal** | 2.55E+00 | 1.40E+00 | 1.64E-01 | -1.95E-02 | -6.12E-01 | 5.42E-01 | -2.06E+00 | -1.98E+00 | 5.13E-02 |
| **Sup.Temporal** | 3.23E-01 | 1.78E-01 | 8.59E-01 | 2.20E-02 | 6.90E-01 | 4.92E-01 | -1.51E+00 | -1.45E+00 | 1.52E-01 |
| **Supramarginal** | 4.73E-01 | 2.60E-01 | 7.95E-01 | 2.09E-02 | 6.57E-01 | 5.13E-01 | -1.64E+00 | -1.57E+00 | 1.21E-01 |
| **Temporal Pole** | -2.64E+00 | -1.64E+00 | 1.03E-01 | 7.78E-03 | 2.14E-01 | 8.31E-01 | NA | NA | NA |
| **Trans. Temporal** | 2.32E+00 | 1.28E+00 | 2.04E-01 | -2.10E-02 | -6.60E-01 | 5.11E-01 | -1.72E+00 | -1.64E+00 | 1.04E-01 |
| **BrainCloud** | | | | | | | | | |
| **Frontal** | -4.18E-01 | -2.38E+00 | 1.76E-02 | 1.83E-03 | 5.29E-01 | 5.97E-01 | 5.07E-01 | 3.72E+00 | 2.26E-04 |
| **BrainEAC** | | | | | | | | | |
| **Frontal** | -2.11E-01 | -5.87E-01 | 5.58E-01 | 2.59E-03 | 5.25E-01 | 6.00E-01 | 8.33E-02 | 4.06E-01 | 6.85E-01 |
| **Occipital** | 4.96E-01 | 1.40E+00 | 1.62E-01 | -7.52E-03 | -1.54E+00 | 1.24E-01 | -8.25E-02 | -4.08E-01 | 6.84E-01 |
| **Temporal** | -4.45E-02 | -1.22E-01 | 9.03E-01 | 1.74E-03 | 3.45E-01 | 7.31E-01 | -8.15E-02 | -3.92E-01 | 6.95E-01 |
| **BrainSpan** | | | | | | | | | |
| **ACC** | -5.29E-01 | -6.91E-01 | 4.91E-01 | 3.52E-02 | 1.09E+00 | 2.78E-01 | -3.43E-01 | -5.36E-01 | 5.93E-01 |
| **DLPFC** | -6.24E-01 | -8.76E-01 | 3.83E-01 | 3.39E-02 | 1.33E+00 | 1.88E-01 | -1.35E-01 | -2.20E-01 | 8.26E-01 |
| **Inferolateral Temporal** | 1.70E-01 | 2.28E-01 | 8.20E-01 | -1.31E-02 | -4.78E-01 | 6.34E-01 | 2.05E-01 | 3.52E-01 | 7.26E-01 |
| **Orbital Frontal** | -1.09E-01 | -1.37E-01 | 8.91E-01 | 2.01E-02 | 6.66E-01 | 5.07E-01 | -7.87E-01 | -1.24E+00 | 2.19E-01 |
| **Posterior Superior Temporal** | -5.45E-02 | -7.70E-02 | 9.39E-01 | 1.09E-02 | 4.30E-01 | 6.68E-01 | -2.89E-01 | -5.05E-01 | 6.15E-01 |
| **Posteroventral Parietal** | 1.03E-01 | 1.38E-01 | 8.90E-01 | 4.36E-03 | 1.60E-01 | 8.73E-01 | -3.65E-01 | -6.26E-01 | 5.33E-01 |
| **Primary Auditory** | 2.19E-01 | 2.91E-01 | 7.72E-01 | -3.59E-03 | -1.30E-01 | 8.97E-01 | -2.79E-01 | -4.62E-01 | 6.45E-01 |
| **Primary Motor** | 4.72E-01 | 5.92E-01 | 5.55E-01 | -9.53E-03 | -3.16E-01 | 7.52E-01 | -5.50E-01 | -8.65E-01 | 3.89E-01 |
| **Primary Somatosensory** | 6.94E-01 | 8.70E-01 | 3.86E-01 | -1.25E-02 | -4.16E-01 | 6.79E-01 | -8.85E-01 | -1.39E+00 | 1.68E-01 |
| **Primary Visual** | 7.48E-02 | 9.95E-02 | 9.21E-01 | -2.38E-03 | -8.58E-02 | 9.32E-01 | -5.00E-02 | -7.89E-02 | 9.37E-01 |
| **Ventrolateral Prefrontal** | -2.32E-01 | -3.27E-01 | 7.44E-01 | 2.36E-02 | 9.30E-01 | 3.55E-01 | -4.73E-01 | -8.05E-01 | 4.23E-01 |
| **GTEx** | | | | | | | | | |
| **ACC** | 8.72E-01 | 1.67E+00 | 9.61E-02 | -1.30E-02 | -1.55E+00 | 1.24E-01 | -1.53E-01 | -6.99E-01 | 4.85E-01 |
| **Frontal** | 6.17E-01 | 1.25E+00 | 2.11E-01 | -1.11E-02 | -1.41E+00 | 1.61E-01 | 3.86E-02 | 1.95E-01 | 8.45E-01 |

**Table S9. *NR3C2* mRNA Expression: Source-Specific Results**

| **Regions** | **Intercept** | | | **Age** | | | | **Sex** | | | |
| --- | --- | --- | --- | --- | --- | --- | --- | --- | --- | --- | --- |
|  | **beta** | **t** | **p** | **beta** | **t** | **p** | | **beta** | **t** | | **p** |
| **Allen Human Brain Atlas** | | | | | | | | | | | |
| **BanksSTS** | -1.70E+00 | -9.90E-01 | 3.24E-01 | 3.91E-02 | 1.04E+00 | | 2.99E-01 | NA | NA | | NA |
| **C.ACC** | 1.81E-01 | 8.43E-02 | 9.33E-01 | 9.75E-03 | 2.58E-01 | | 7.97E-01 | -7.15E-01 | -5.78E-01 | | 5.65E-01 |
| **C.Mid.Frontal** | -1.24E+00 | -5.78E-01 | 5.65E-01 | 1.78E-02 | 4.71E-01 | | 6.39E-01 | 5.84E-01 | 4.72E-01 | | 6.38E-01 |
| **Cuneus** | 2.50E+00 | 1.16E+00 | 2.49E-01 | -4.27E-02 | -1.13E+00 | | 2.61E-01 | -8.20E-01 | -6.62E-01 | | 5.09E-01 |
| **Entorhinal** | -1.45E+00 | -6.76E-01 | 5.01E-01 | 1.94E-02 | 5.13E-01 | | 6.09E-01 | 7.56E-01 | 6.11E-01 | | 5.43E-01 |
| **Frontal Pole** | -6.08E+00 | -2.79E+00 | 6.09E-03 | 1.52E-01 | 2.70E+00 | | 7.87E-03 | NA | NA | | NA |
| **Fusiform** | -3.95E-01 | -1.83E-01 | 8.55E-01 | 3.09E-02 | 8.19E-01 | | 4.15E-01 | -1.10E+00 | -8.91E-01 | | 3.75E-01 |
| **Inf.Parietal** | -2.67E-01 | -1.24E-01 | 9.01E-01 | 4.34E-03 | 1.15E-01 | | 9.09E-01 | 9.93E-02 | 8.02E-02 | | 9.36E-01 |
| **Inf.Temporal** | -1.87E+00 | -8.71E-01 | 3.86E-01 | 5.35E-02 | 1.42E+00 | | 1.60E-01 | -4.79E-01 | -3.87E-01 | | 6.99E-01 |
| **Insula** | -1.90E+00 | -8.84E-01 | 3.79E-01 | 4.79E-02 | 1.27E+00 | | 2.07E-01 | -1.63E-01 | -1.32E-01 | | 8.95E-01 |
| **Isthmus Cingulate** | -2.43E+00 | -1.13E+00 | 2.63E-01 | 3.09E-02 | 8.19E-01 | | 4.15E-01 | 1.33E+00 | 1.08E+00 | | 2.84E-01 |
| **L.Occipital** | -1.65E+00 | -7.67E-01 | 4.45E-01 | 4.12E-02 | 1.09E+00 | | 2.78E-01 | -1.22E-01 | -9.84E-02 | | 9.22E-01 |
| **L.Orbitofrontal** | 2.95E+00 | 1.37E+00 | 1.74E-01 | -6.91E-02 | -1.83E+00 | | 7.05E-02 | -1.32E-02 | -1.07E-02 | | 9.91E-01 |
| **Lingual** | -7.70E-01 | -3.58E-01 | 7.21E-01 | 1.94E-02 | 5.13E-01 | | 6.09E-01 | -6.36E-02 | -5.14E-02 | | 9.59E-01 |
| **M.Orbitofrontal** | -1.69E+00 | -7.85E-01 | 4.35E-01 | 4.43E-02 | 1.17E+00 | | 2.44E-01 | -2.31E-01 | -1.87E-01 | | 8.52E-01 |
| **Mid.Temporal** | -9.05E-01 | -4.21E-01 | 6.75E-01 | 3.73E-02 | 9.87E-01 | | 3.26E-01 | -8.14E-01 | -6.57E-01 | | 5.13E-01 |
| **P.Cingulate** | 6.26E-01 | 2.91E-01 | 7.72E-01 | 9.65E-03 | 2.56E-01 | | 7.99E-01 | -1.24E+00 | -1.00E+00 | | 3.18E-01 |
| **Paracentral** | -4.32E-01 | -2.01E-01 | 8.41E-01 | 2.93E-02 | 7.77E-01 | | 4.39E-01 | -9.77E-01 | -7.89E-01 | | 4.32E-01 |
| **Parahippocampal** | -1.54E+00 | -5.96E-01 | 5.52E-01 | 2.11E-02 | 4.43E-01 | | 6.59E-01 | -6.68E-01 | -4.95E-01 | | 6.22E-01 |
| **Pars Opercularis** | -6.68E-01 | -3.10E-01 | 7.57E-01 | 4.76E-03 | 1.26E-01 | | 9.00E-01 | 5.58E-01 | 4.51E-01 | | 6.53E-01 |
| **Pars Orbitalis** | 1.11E+00 | 7.48E-01 | 4.56E-01 | -3.57E-02 | -1.03E+00 | | 3.03E-01 | NA | NA | | NA |
| **Pars Triangularis** | 3.66E+00 | 1.35E+00 | 1.79E-01 | -1.82E-02 | -3.62E-01 | | 7.18E-01 | 9.42E-02 | 7.60E-02 | | 9.40E-01 |
| **Pericalcarine** | 1.27E+00 | 5.92E-01 | 5.55E-01 | -5.95E-03 | -1.58E-01 | | 8.75E-01 | -1.22E+00 | -9.89E-01 | | 3.25E-01 |
| **Post Central** | -1.43E+00 | -6.63E-01 | 5.09E-01 | 3.47E-02 | 9.18E-01 | | 3.61E-01 | -5.77E-02 | -4.66E-02 | | 9.63E-01 |
| **Precentral** | 4.40E-01 | 2.05E-01 | 8.38E-01 | -2.01E-02 | -5.34E-01 | | 5.95E-01 | 4.99E-01 | 4.03E-01 | | 6.88E-01 |
| **Precuneus** | 2.75E+00 | 1.28E+00 | 2.04E-01 | -6.65E-02 | -1.76E+00 | | 8.15E-02 | 8.93E-02 | 7.22E-02 | | 9.43E-01 |
| **R.ACC** | -1.01E+00 | -4.72E-01 | 6.38E-01 | 3.49E-02 | 9.24E-01 | | 3.58E-01 | -5.63E-01 | -4.55E-01 | | 6.50E-01 |
| **R.Mid.Frontal** | -1.17E+00 | -5.42E-01 | 5.89E-01 | 1.86E-02 | 4.93E-01 | | 6.23E-01 | 4.50E-01 | 3.63E-01 | | 7.17E-01 |
| **Sup.Frontal** | -1.33E+00 | -6.19E-01 | 5.37E-01 | 3.79E-02 | 1.00E+00 | | 3.18E-01 | -3.34E-01 | -2.70E-01 | | 7.88E-01 |
| **Sup.Parietal** | 2.04E+00 | 9.48E-01 | 3.46E-01 | -4.04E-02 | -1.07E+00 | | 2.87E-01 | -3.86E-01 | -3.12E-01 | | 7.56E-01 |
| **Sup.Temporal** | -2.17E+00 | -1.01E+00 | 3.17E-01 | 4.85E-02 | 1.28E+00 | | 2.02E-01 | 1.27E-01 | 1.03E-01 | | 9.18E-01 |
| **Supramarginal** | -1.44E+00 | -6.71E-01 | 5.04E-01 | 3.81E-02 | 1.01E+00 | | 3.15E-01 | -2.15E-01 | -1.74E-01 | | 8.63E-01 |
| **Temporal Pole** | -3.52E+00 | -2.30E+00 | 2.29E-02 | 2.78E-02 | 8.02E-01 | | 4.24E-01 | NA | NA | | NA |
| **Trans. Temporal** | -1.07E+00 | -4.99E-01 | 6.19E-01 | 1.49E-02 | 3.96E-01 | | 6.93E-01 | 5.26E-01 | 4.25E-01 | | 6.72E-01 |
| **BrainCloud** | | | | | | | | | | | |
| **Frontal** | -1.36E+00 | -7.72E+00 | 9.16E-14 | 3.22E-02 | 9.33E+00 | 7.06E-19 | | 2.75E-01 | | 2.02E+00 | 4.43E-02 |
| **BrainEAC** | | | | | | | | | | | |
| **Frontal** | -2.11E-01 | -5.87E-01 | 5.58E-01 | 2.59E-03 | 5.25E-01 | 6.00E-01 | | 8.33E-02 | 4.06E-01 | | 6.85E-01 |
| **Occipital** | 4.96E-01 | 1.40E+00 | 1.62E-01 | -7.52E-03 | -1.54E+00 | 1.24E-01 | | -8.25E-02 | -4.08E-01 | | 6.84E-01 |
| **Temporal** | -4.45E-02 | -1.22E-01 | 9.03E-01 | 1.74E-03 | 3.45E-01 | 7.31E-01 | | -8.15E-02 | -3.92E-01 | | 6.95E-01 |
| **BrainSpan** | | | | | | | | | | | |
| **ACC** | -8.02E-01 | -1.13E+00 | 2.61E-01 | 5.36E-02 | 1.80E+00 | 7.56E-02 | | -5.30E-01 | -8.94E-01 | | 3.73E-01 |
| **DLPFC** | -1.06E+00 | -1.60E+00 | 1.13E-01 | 4.83E-02 | 2.04E+00 | 4.36E-02 | | 1.38E-01 | 2.43E-01 | | 8.08E-01 |
| **Inferolateral Temporal** | -7.24E-01 | -1.05E+00 | 2.97E-01 | 2.12E-02 | 8.37E-01 | 4.05E-01 | | 5.00E-01 | 9.26E-01 | | 3.57E-01 |
| **Orbital Frontal** | -6.47E-01 | -8.77E-01 | 3.83E-01 | 4.17E-02 | 1.49E+00 | 1.38E-01 | | -7.12E-01 | -1.21E+00 | | 2.30E-01 |
| **Posterior Superior Temporal** | -8.84E-01 | -1.35E+00 | 1.80E-01 | 5.35E-02 | 2.28E+00 | 2.45E-02 | | -3.45E-01 | -6.50E-01 | | 5.17E-01 |
| **Posteroventral Parietal** | -5.14E-01 | -7.45E-01 | 4.58E-01 | 3.35E-02 | 1.32E+00 | 1.88E-01 | | -3.81E-01 | -7.05E-01 | | 4.83E-01 |
| **Primary Auditory** | -3.66E-01 | -5.25E-01 | 6.00E-01 | 1.87E-02 | 7.29E-01 | 4.67E-01 | | -9.14E-02 | -1.63E-01 | | 8.71E-01 |
| **Primary Motor** | -1.64E-01 | -2.22E-01 | 8.25E-01 | 1.62E-02 | 5.81E-01 | 5.62E-01 | | -4.70E-01 | -7.97E-01 | | 4.27E-01 |
| **Primary Somatosensory** | 1.27E-01 | 1.71E-01 | 8.64E-01 | 7.63E-03 | 2.73E-01 | 7.85E-01 | | -6.69E-01 | -1.14E+00 | | 2.59E-01 |
| **Primary Visual** | 1.44E-01 | 2.07E-01 | 8.36E-01 | -6.15E-03 | -2.40E-01 | 8.11E-01 | | -2.08E-02 | -3.55E-02 | | 9.72E-01 |
| **Ventrolateral Prefrontal** | -9.86E-01 | -1.50E+00 | 1.37E-01 | 5.37E-02 | 2.28E+00 | 2.46E-02 | | -2.25E-01 | -4.13E-01 | | 6.81E-01 |
| **GTEx** | | | | | | | | | | | |
| **ACC** | 1.45E+00 | 2.91E+00 | 4.02E-03 | -2.77E-02 | -3.43E+00 | 7.32E-04 | | 2.48E-01 | 1.18E+00 | | 2.38E-01 |
| **Frontal** | 9.07E-01 | 1.92E+00 | 5.56E-02 | -2.01E-02 | -2.66E+00 | 8.32E-03 | | 3.75E-01 | 1.98E+00 | | 4.87E-02 |

**Table S10. Results of GO Enrichment Analysis**

| **GO Group** | **Overlap** | **Set Size** | **P.Value** | **adj. P.Value** | **Expected** | **Aspect** |
| --- | --- | --- | --- | --- | --- | --- |
| intracellular ligand-gated ion channel activity | 7 | 10 | 4.85E-04 | 1 | 3.81 | MF |
| myelin sheath | 12 | 26 | 1.01E-03 | 1 | 2.51 | CC |
| vacuole | 31 | 100 | 1.24E-03 | 1 | 1.69 | CC |
| phosphatidylinositol bisphosphate binding | 8 | 15 | 2.34E-03 | 1 | 2.90 | MF |
| regulation of localization | 122 | 542 | 3.04E-03 | 1 | 1.23 | BP |
| ligand-gated cation channel activity | 11 | 26 | 3.83E-03 | 1 | 2.30 | MF |
| phosphatidylinositol-4,5-bisphosphate binding | 6 | 10 | 4.00E-03 | 1 | 3.27 | MF |
| bone remodeling | 8 | 17 | 6.26E-03 | 1 | 2.56 | BP |
| transmembrane receptor protein tyrosine kinase activity | 10 | 24 | 6.59E-03 | 1 | 2.27 | MF |
| inorganic anion transport | 15 | 43 | 7.02E-03 | 1 | 1.90 | BP |
| MF: molecular function, CC: cellular component, BP: biological process | | | | | | |

**Figure S6**. Average Gene-Thickness Similarity by Age Group. Average *NR3C1*-Thickness Similarity is plotted as a dotted red-line while the average *NR3C2*-Thickness Similarity is plotted as a solid red-line. A) Distribution of 2,511 average Gene-Thickness similarities for participants <27 years old (n=8,080). B) Distribution of 2,511 average Gene-Thickness similarities for participants between 27 and 60 years old (inclusive; n=9,171) C) Distribution of 2,511 average Gene-Thickness similarities for participants >60 years old (n=12,892).

**Supplementary References**

Ardlie KG, DeLuca DS, Segrè A V., Sullivan TJ, Young TR, Gelfand ET, Trowbridge CA, Maller JB, Tukiainen T, Lek M, Ward LD, Kheradpour P, Iriarte B, Meng Y, Palmer CD, Esko T, Winckler W, Hirschhorn JN, Kellis M, MacArthur DG, Getz G, Shabalin AA, Li G, Zhou YH, Nobel AB, Rusyn I, Wright FA, Lappalainen T, Ferreira PG, Ongen H, Rivas MA, Battle A, Mostafavi S, Monlong J, Sammeth M, Melé M, Reverter F, Goldmann JM, Koller D, Guigó R, McCarthy MI, Dermitzakis ET, Gamazon ER, Im HK, Konkashbaev A, Nicolae DL, Cox NJ, Flutre T, Wen X, Stephens M, Pritchard JK, Tu Z, Zhang B, Huang T, Long Q, Lin L, Yang J, Zhu J, Liu J, Brown A, Mestichelli B, Tidwell D, Lo E, Salvatore M, Shad S, Thomas JA, Lonsdale JT, Moser MT, Gillard BM, Karasik E, Ramsey K, Choi C, Foster BA, Syron J, Fleming J, Magazine H, Hasz R, Walters GD, Bridge JP, Miklos M, Sullivan S, Barker LK, Traino HM, Mosavel M, Siminoff LA, Valley DR, Rohrer DC, Jewell SD, Branton PA, Sobin LH, Barcus M, Qi L, McLean J, Hariharan P, Um KS, Wu S, Tabor D, Shive C, Smith AM, Buia SA, Undale AH, Robinson KL, Roche N, Valentino KM, Britton A, Burges R, Bradbury D, Hambright KW, Seleski J, Korzeniewski GE, Erickson K, Marcus Y, Tejada J, Taherian M, Lu C, Basile M, Mash DC, Volpi S, Struewing JP, Temple GF, Boyer J, Colantuoni D, Little R, Koester S, Carithers LJ, Moore HM, Guan P, Compton C, Sawyer SJ, Demchok JP, Vaught JB, Rabiner CA, Lockhart. 2015. The Genotype-Tissue Expression (GTEx) pilot analysis: Multitissue gene regulation in humans. Science. 348:648–660.

Colantuoni C, Lipska BK, Ye T, Hyde TM, Weinberger DR, Kleinman JE. 2012. Temporal dynamics and genetic control of transcription in the human prefrontal cortex. Nature. 478:519–523.

Collado-Torres L, Nellore A, Kammers K, Ellis SE, Taub MA, Hansen KD, Jaffe AE, Langmead B, Leek JT. 2017. Reproducible RNA-seq analysis using recount2. Nat Biotechnol. 35:319–321.

Hawrylycz MJ, Lein ES, Guillozet-Bongaarts AL, Shen EH, Ng L, Miller JA, Lagemaat LN van de, Smith KA, Ebbert A, Riley ZL, Abajian C, Beckmann CF, Bernard A, Bertagnolli D, Boe AF, Cartagena PM, Chakravarty MM, Chapin M, Chong J, Dalley RA, Daly BD, Dang C, Datta S, Dee N, Dolbeare TA, Faber V, Feng D, Fowler DR, Goldy J, Gregor BW, Haradon Z, Haynor DR, Hohmann JG, Horvath S, Howard RE, Jeromin A, Jochim JM, Kinnunen M, Lau C, Lazarz ET, Lee C, Lemon TA, Li L, Li Y, Morris JA, Overly CC, Parker PD, Parry SE, Reding M, Royall JJ, Schulkin J, Sequeira PA, Slaughterbeck CR, Smith SC, Sodt AJ, Sunkin SM, Swanson BE, Vawter MP, Williams D, Wohnoutka P, Zielke HR, Geschwind DH, Hof PR, Smith SM, Koch C, Grant SGN, Jones AR. 2012. An anatomically comprehensive atlas of the adult human brain transcriptome. Nature. 489:391–399.

Trabzuni D, Ryten M, Walker R, Smith C, Imran S, Ramasamy A, Weale ME, Hardy J. 2011. Quality control parameters on a large dataset of regionally dissected human control brains for whole genome expression studies. J Neurochem. 119:275–282.
